# The effects of vitamin D supplementation on inflammatory biomarkers in patients with asthma: a systematic review and meta-analysis of randomized controlled trials

**DOI:** 10.3389/fimmu.2024.1335968

**Published:** 2024-03-13

**Authors:** Asmae El Abd, Harika Dasari, Philippe Dodin, Helen Trottier, Francine M. Ducharme

**Affiliations:** ^1^ Sainte-Justine University Health Center, Research Center, Montreal, QC, Canada; ^2^ Department of Social and Preventive Medicine, School of Public Health, University of Montreal, Montreal, QC, Canada; ^3^ Department of Pediatrics, Faculty of Medicine, University of Montreal, Sainte-Justine Hospital, Montreal, QC, Canada

**Keywords:** inflammation, asthma, vitamin D, biomarkers, systematic review, meta-analysis

## Abstract

**Background:**

While the association between vitamin D and several inflammatory biomarkers in asthma patients has been extensively reported, it remains unclear whether supplementation modifies these biomarkers. This review aims to evaluate the impact of vitamin D supplementation on inflammatory biomarkers measured *in vivo* in individuals with asthma.

**Methods:**

We conducted a systematic review of randomized controlled trials (RCTs) published until November 2022 in six electronic databases evaluating the impact of vitamin D supplementation (any dose, form, administration route, frequency, or duration) compared to placebo in children or adults. The two co-primary outcomes were serum IgE and blood eosinophils reported at the endpoint. Secondary outcomes included other markers of type 2 inflammation (e.g., sputum eosinophils, fractional exhaled nitric oxide, etc.), anti-inflammatory biomarkers (e.g., interleukin (IL)-10, etc.), markers of non-type 2 inflammation (e.g., high-sensitivity C-reactive protein, etc.), and non-specific biomarkers (e.g., macrophages, etc.). Data were aggregated using fixed or random effect models.

**Results:**

Thirteen RCTs (5 in adults, 5 in pediatric patients, and 3 in mixed age groups) testing doses of vitamin D supplementation ranging from 800 to 400,000 IU over periods of 6 weeks to 12 months were included. Eight studies provided data on serum IgE and four on blood eosinophils. As secondary outcomes, three studies reported on sputum eosinophils, four on FeNO, five on serum IL-10, and two on airway IL-10. Compared to placebo, vitamin D supplementation had no significant effect on serum IgE (Mean difference [MD] [95% CI]: 0.06 [-0.13, 0.26] IU/mL), blood eosinophils (MD [95% CI]: - 0.02 [-0.11, 0.07] 10^3^/μL), or FeNO (MD [95% CI]: -4.10 [-10.95, 2.75] ppb) at the endpoint. However, the vitamin D supplementation group showed higher serum IL-10 levels compared to placebo (MD [95% CI]: 18.85 [1.11, 36.59] pg/ml) at the endpoint. Although data could not be aggregated, narrative synthesis suggested no significant effect of supplementation on sputum eosinophils and IL-10 in both sputum and exhaled breath condensate, at the endpoint.

**Conclusion:**

Vitamin D supplementation in individuals with asthma was not associated with lower inflammatory biomarkers related to type 2 inflammation. However, it was significantly associated with higher serum IL-10 compared to placebo.

**Systematic review registration:**

https://www.crd.york.ac.uk/PROSPERO/, identifier CRD42022365666.

## 1 Introduction

Suboptimal level of serum 25-hydroxyvitamin D (25(OH)D) has been associated with an elevated risk of hospitalization, emergency department visits, exacerbations, and use of rescue oral corticosteroids (OCS) for asthma in both adults and children (1–3). A 2016 systematic review and meta-analysis of five randomized controlled trials (RCTs) concluded that vitamin D supplementation reduced by 36% the risk of asthma exacerbations requiring the use of oral corticosteroids (OCS); it also decreased by 61% the risk of individuals experiencing at least one exacerbation necessitating an emergency department visit, hospitalization, or both (4). While an updated 2023 systematic review and meta-analysis of 20 RCTs revealed that vitamin D supplementation was no longer associated with improved asthma control, it highlighted significant heterogeneity and an evidence gap in patients with severe asthma and those with vitamin D deficiency (5). In the meanwhile, findings of the 2016 review had generated various hypotheses regarding the mechanisms of action through which vitamin D could theoretically influence asthma exacerbations, notably by targeting inflammation, a central pathological feature of asthma (6).

Indeed, evidence suggests that vitamin D can modulate inflammation in individuals with inflammatory diseases by interacting with numerous immune and inflammatory cells and regulating various pro- and anti-inflammatory biomarkers. Recent reviews have reported that vitamin D regulates the functions of monocytes and macrophages by increasing the production of antimicrobial peptides, such as cathelicidin (LL-37) and defensing β2 (7, 8). Additionally, vitamin D promotes anti-inflammatory effects in macrophages by boosting interleukin (IL)-10 production and reducing the production of pro-inflammatory cytokines, including IL-1, IL-6, and tumor necrosis factor-alpha (TNF-α) (7–10). Vitamin D also inhibits cytokine production by dendritic cells, resulting in decreased IL-12 production and an enhanced induction of regulatory T cells (Tregs) and IL-10 production (7–9, 11, 12). Moreover, some studies suggest that vitamin D suppresses B lymphocyte differentiation, curtails their proliferation, reduces immunoglobulin (Ig) production, and enhances IL-10 production, thereby exerting an additional regulatory effect (7, 8). Furthermore, vitamin D plays a pivotal role in modulating the immune system by directly affecting the differentiation of T lymphocytes into various T helper (Th) cells, including Th1, Th2, and Th17, and by modulating their interactions with other immune cells (7, 9). This leads to a decreased proliferation of Th1 cells and a reduction in pro-inflammatory cytokine such as IL-2, interferon-gamma (IFN-γ), and TNF-α (7, 9). It also reduces pro-inflammatory cytokines associated with Th17 cells, including IL-17, and enhances the activation of Th2 cells along with the secretion of anti-inflammatory cytokines like IL-10 (7, 9). However, the effects of vitamin D on Th2 cells in patients with asthma show inconsistency across studies. A recent review indicates that vitamin D supplementation may rather reduce the Th2 response and associated cytokines in asthma by boosting Treg cells and elevating IL-10 levels (13).

Consequently, several inflammatory biomarkers have been integrated into subsequent asthma research studies, including those involving vitamin D interventions (14), and a considerable amount of data elucidating potential inflammatory mechanisms has been collected. Particular emphasis has been given to biomarkers commonly measured in clinical practice, such as serum IgE, serum and sputum eosinophils, and fractional exhaled nitric oxide (FeNO) which are indicators of type 2 inflammation (15, 16), as well as anti-inflammatory biomarkers like IL-10 (17). Five systematic reviews and meta-analyses evaluated the impact of vitamin D supplementation in asthma on those inflammatory biomarkers (5, 18–21). These reviews often included studies with *ex vivo* experimental designs, pre-post studies without an independent control group, and excluded trials with short follow-up durations (less than 12 weeks). Furthermore, they did not distinguish between the various types of biological samples used to measure these inflammatory biomarkers (e.g., sputum, serum, and exhaled breath condensate), and the range of biomarkers considered in their search strategy was limited, preventing the identification of other studies evaluating other relevant inflammatory biomarkers. Therefore, a more comprehensive systematic review and meta-analysis of all relevant vitamin supplementation RCTs, examining various inflammatory biomarkers from different biological compartments, is of interest. This review aimed to assess the effects of vitamin D supplementation, compared to a placebo, on inflammatory biomarkers measured *in vivo* in patients of all ages with asthma.

## 2 Materials and methods

The study protocol was registered in International Prospective Register of Systematic Reviews (PROSPERO) (registration number: CRD42022365666). Guidelines outlined by the Preferred Reporting Items for Systematic Review and Meta-analysis (PRISMA) were followed (22).

### 2.1 Search strategy and selection criteria

A systematic search of relevant trials, published in either French or English until 24^th^ November 2022, was performed on six databases: PubMed, Medline, All EBM (Evidence-Based Medicine), Embase, CINAHL (Cumulative Index to Nursing and Allied Health Literature), and Web of Science. This task was performed by a librarian (PD) with special training and skills in literature searches. The full search strategy including MESH (Medical Subject Headings) terms and keywords used for each database is detailed in **Supplementary Method S1**. Additionally, we reviewed the bibliography of a 2023 systematic review of RCTs (5) that examined the clinical impact of vitamin D supplementation on asthma to identify any further trials. To be eligible for inclusion, studies had to meet the following PICOS criteria:

Population: Human individuals diagnosed with asthma, irrespective of age (including children and adults), gender, ethnicity or body mass index (BMI).

Interventions: Vitamin D supplementation, regardless of the dose, form, administration route (oral or non-oral), frequency (e.g., single doses, daily, weekly, etc.), or duration.

Comparator: Placebo or no vitamin D supplementation, as the control group.

Co-intervention(s): Standard asthma therapy as allowed co-intervention, only if similarly provided to both groups. Of note, vitamin D supplements provided in combination with any other vitamin, antioxidants, or mineral supplements were excluded.

Outcomes: The two *a priori* specified co-primary outcomes were serum IgE and blood eosinophils. For secondary outcomes, we considered other inflammatory biomarkers in various biological fluids, including: (i) pro-inflammatory biomarkers of type 2 inflammation, such as sputum eosinophils, FeNO, serum eosinophil cationic protein (ECP), as well as IL-4, IL-5, and IL-13; (ii) pro-inflammatory biomarkers of non-type 2 inflammation, such as high-sensitivity C-reactive protein (HsCRP); (iii) anti-inflammatory biomarkers, such as IL-10; (iv) non-specific biomarkers whose roles as pro- or anti-inflammatory biomarkers are unclear or variable, such as macrophages.

Study Design: Randomized, placebo-controlled, open-label or double-blind, parallel-group design trials. Of note, crossover trials were excluded.

### 2.2 Study selection and data extraction

Two reviewers (AEA and HD) independently screened titles and abstracts for all identified articles, and the full text for all abstracts deemed potentially relevant. Patient’s characteristics (age, gender, asthma status, duration of disease and BMI), study methodology (i.e., design, eligibility, intervention, exposure (e.g., food intake), vitamin levels, and outcomes), and results were extracted independently by two reviewers (AEA and HD) using a predesigned form. All conflicts during study selection and data extraction were resolved by discussion; a third independent reviewer (FMD) was consulted in cases of disagreement. The Covidence systematic review software, Veritas Health Innovation, Melbourne, Australia, was used to manage and streamline the process.

### 2.3 Quality assessment

Two independent reviewers (AEA and HD) evaluated the methodological quality of eligible studies. Cochrane Handbook risk of bias tool 2 (23) was used to assess the risk of bias based on several domains, namely that arising from (i) randomization process, (ii) deviations from intended interventions, (iii) missing outcome data, (iv) outcome measurement, (v) selective reporting of results. Each study was categorized as being at low, unclear, or high risk of bias. Any disagreement regarding the study’s methodological quality was resolved by reaching a consensus or obtaining input from a third reviewer (FMD).

### 2.4 Quantitative analysis

Meta-analyses were performed using the Review Manager (RevMan) computer program (Version 5.4.1, London, United Kingdom, The Cochrane Collaboration, 2020). The mean difference with 95% confidence interval (CI) was reported at the endpoint. When results were reported as a median and interquartile range (IQR), we transformed them into mean and standard deviation (SD) if the data were symmetrical following the method of Luo et al. (2018) (24) and Wan et al. (2014) (25). For inflammatory biomarkers measured at different time points, we considered the latest time point after randomization. Appreciable heterogeneity was assumed if I^2^ was higher than 50 and the P was less than 0.1 (26). The meta-analyses were performed using an inverse variance with fixed effect model if I^2^ was inferior to 0.50, otherwise a random effect model was used.

Predefined subgroup analyses for all outcomes included: age group (children vs. adults), mean baseline vitamin D status [serum 25(OH)D level <25 nmol/L (indicating deficiency) vs. 25-50 nmol/L (indicating insufficiency) vs. ≥50 nmol/L (indicating sufficiency)], frequency of administration (single dose vs. daily doses vs. weekly doses vs. single or repeated boluses (≥30,000 IU) with or without daily doses), and form of vitamin D administered (cholecalciferol vs. calcidiol vs. calcitriol). Two sensitivity analyses were performed for all co-primary outcomes: first, by excluding studies with an unclear risk of bias; and second, by excluding studies with results reported as median (IQR) that were transformed into mean (SD) for the main analysis. For studies that could not be included in the meta-analysis, results were summarized either narratively in tabulated form with the direction of observed effect, or by providing any available quantitative results at the endpoint.

## 3 Results

### 3.1 Search results

**Figure 1:**
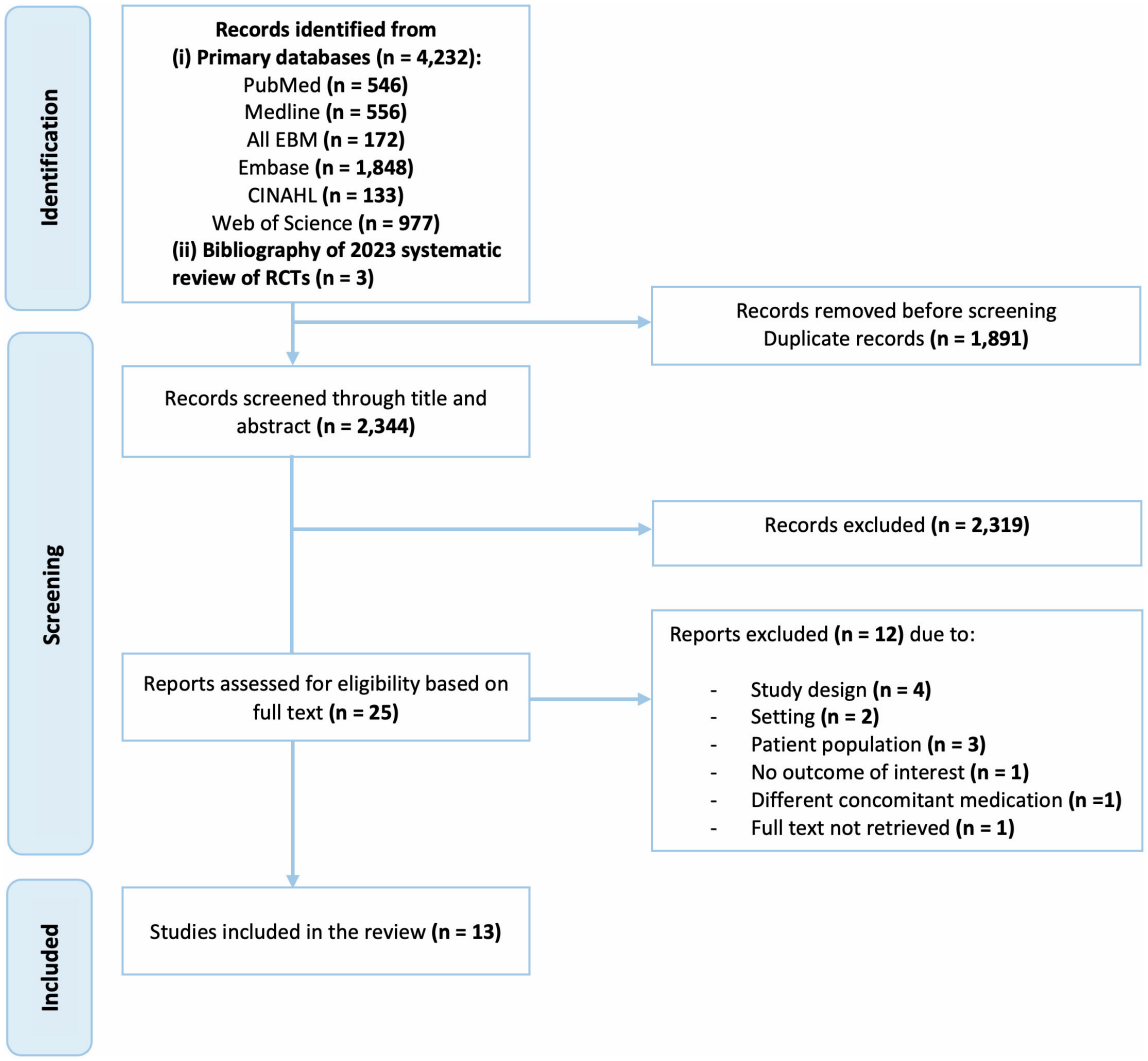
Selection process for eligible studies from all identified citations.

The literature search yielded 4,232 citations. After eliminating 1,891 duplicates, 2,344 citations were screened based on their titles and abstracts, and 25 full-text articles were selected for further assessment. A total of 12 RCTs were considered not eligible, resulting in 13 RCTs included in the review (**Figure 1**).

### 3.2 Study characteristics

In the 13 eligible RCTs (27–39), 1459 (735 intervention: 724 control) participants were randomized (**Table 1**). Five RCTs were conducted exclusively in children (29, 33, 36, 38, 39), five in adults (28, 30, 31, 35, 37), and three in mixed age groups (27, 32, 34). Asthma severity was reported in only four RCTs. One trial recruited only patients with mild asthma (29), while another focused on those with moderate persistent asthma (39). The remaining two trials reported that the majority of participants had moderate persistent asthma (28) or severe asthma (37). Asthma duration was reported in only seven studies, with durations that ranged from 4 years (31) to 25 years (34). Four trials reported average BMIs below 25 kg/m² (29, 33, 38, 39), while six reported average BMIs of 25 kg/m² or higher (27, 28, 30, 31, 36, 37), suggesting normal weight vs. overweight and obese, respectively. In one trial, 26% of participants had a BMI over 30 kg/m² (34). The remaining two trials failed to report BMI (32, 35). The mean baseline vitamin D status indicated deficiency (25(OH)D <25 nmol/L) in one trial (27), insufficiency (25(OH) <50 nmol/L) in seven trials (27, 28, 30, 32, 34, 37, 39), sufficiency (25(OH)D ≥50 nmol/L) in five trials (29, 31, 33, 36, 38), and one RCT failed to report mean baseline 25(OH)D (35). The status of other vitamins was not described in the included studies. Both dose and frequency of administration of supplemental vitamin D varied among included trials. Only one three-month RCT administered a single dose of 2,000 IU (27). Five RCTs (33, 35, 36, 38, 39) tested daily doses ranging from 800 to 4,000 IU, with study durations ranging from 3 to 6 months. Weekly doses ranging from 14,000 to 60,000 IU were examined in three RCTs (28, 29, 32) that lasted from 6 weeks to 6 months. Finally, four RCTs administered boluses: two separate RCTs used a single bolus of at least 30,000 IU, one lasting 9 weeks (31) and the other 3 months (37); a 12-month RCT administered a bi-monthly bolus of 120,000 IU (34); and an additional RCT in adults combined a single 100,000 IU bolus with a daily dose of 4,000 IU (30). The dietary patterns or specific foods consumed by the participants, including any recommendations for taking vitamin D with a fat-rich meal, were not specified in any of the included studies.

**Table 1:** Summary of characteristics and outcomes of included studies examining the impact of vitamin D supplementation on inflammatory biomarkers in subjects with asthma.

Reference, Year	Design, Setting	Subject N (age, % male, asthma severity)	Duration of asthma (Years)		BMI (kg/m^2^)		Frequency (duration of intervention)	Treatment Arms	Mean baseline serum 25OHD (nmol/L)		Follow-up timepoints	Effects on inflammatory biomarkers*
			Intervention	Control	Intervention	Control			Intervention	Control		
**Abbas et al.** (27)**, 2017**	RCT, Iraq	44 (14-71 years old, male = 25%, not specified)	20.2 ± 15.4	12.8 ± 11.2	29.25 ± 7.44	33.20 ± 8.54	Single dose	**Intervention**: vitamin D (cholecalciferol) (2,000 IU) + conventional asthma therapy (n = 24) **Control**: no vitamin D supplements + conventional asthma therapy (n = 20)	15.8 ± 11.6	22.2 ± 17.0	3 months	**↑** Serum concentrations of IL-10 **↔** Serum concentrations of TNF-α
**Andújar-Espinosa et al.** (28)**, 2021**	RCT, Spain	112 (≥ 18, 55 ± 15.4 years old, male = 22%, Intermittent: 17 (15.2%) - Mild Persistent: 20 (17.9%) - Moderate Persistent: 56 (50.0%) - Severe Persistent: 19 (17.0%))	21.29 ± 11.30	18.61 ± 9.25	28.21 ± 5.23	29.83 ± 7.41	Weekly (not specified)	**Intervention**: vitamin D (calcidiol) (16,000 IU)/week (n = 56) **Control**: placebo (n = 56)	41.8 ± 16.7	43.7 ± 14.3	6 months	**↔** Serum IgE
**BarYoseph et al.** (29)**, 2015**	RCT, Israel	39 (6-18 years old, male = 64.1%, mild asthma)	Not specified	Not specified	19.38 ± 3.29	21.53 ± 3.79	Weekly (6 weeks)	**Intervention:** vitamin D (cholecalciferol) (14,000 IU/week) (n = 20) **Control:** placebo (n = 19)	51.9 ± 16.2	49.9 ± 17.7	6 weeks	**↔** Blood eosinophils, serum IgE and serum Hs-CRP, FeNO, and airway IL-4 (EBC) **↑** Airway IL-5, IL-10 and IFN-γ (EBC) **↓** Airway IL-17 (EBC)
**Castro et al.** (30)**, 2014**	RCT, USA	408 (≥ 18, 39.7 ± years old, male = 31.9%, not specified)	24.9 ± 13.5	25.0 ± 12.8	32.0 ± 8.19	31.53 ± 9.51	Single bolus + daily (28 weeks)	**Intervention:** vitamin D (cholecalciferol) (100,000 IU once and 4,000 IU/day) + inhaled ciclesonide (n = 201) **Control:** placebo + inhaled ciclesonide (n = 207)	49.7 (36.2, 62.4)	46.9 (33.4, 59.2)	28 weeks	**↔** Sputum eosinophils
**De Groot et al.** (31)**, 2015**	RCT, Netherlands	44 (18-73 years old, male = 59.1%, not specified)	4 (3, 30)	5 (2, 16)	26.6 ± 4.2	26.9 ± 4.8	Single bolus	**Intervention:** vitamin D (cholecalciferol) (400,000 IU) (n = 22) **Control:** placebo (n = 22)	60.0 (47.0, 78.0)	57.0 (40.0, 70.0)	9 weeks	**↔** Sputum and blood eosinophils and neutrophils, serum IgE and FeNO
**Dodamani et al.** (32)**, 2019**	RCT, India	30 (≥ 12, 32.8 ± 12.1 years old, male = 70%, not specified)	8 (0.3 - 25)	7 (0.7 - 25)	Not specified	Not specified	Weekly (8 weeks)	**Intervention:** vitamin D (cholecalciferol) (60,000 IU/Week) **+** prednisolone (n = 15) **Control:** prednisolone (n = 15)	53.7 (15.2 - 103.8)	40.9 (13.5 - 102.6)	2, 4 and 6 months	**↔** Serum IgE, IL-4, IL-6, IL-10, and IL-17
**Kerley et al.** (33)**, 2016**	RCT, Ireland	44 (6-16 years old, male = 52.3%, not specified)	Not specified	Not specified	19.6 (17.0, 22.0)	18.2 (16.0, 20.0)	Daily (15 weeks)	**Intervention:** vitamin D (cholecalciferol) (2,000 IU/day) (n = 19) **Control:** Placebo (n = 25)	58.0 (39.0, 69.0)	51.0 (39.0, 64.0)	15 weeks	**↔** Serum LL-37, IL-10, IgE, IgA, ECP and blood eosinophils **↑** Serum Hs-CRP
**Martineau et al.** (34)**, 2015**	RCT, UK	250 (16-80 years old, male = 43.6%, not specified)	25 (14, 36)	23 (14, 32)	**(≥30):** 36 (29.0%)	**(≥30):** 29 (23.0%)	Monthly (1 year)	**Intervention:** vitamin D (cholecalciferol) (120,000 IU/2 months) + (n = 125) **Control:** Placebo (n = 125)	49.8 ± 25.2	49.4 ± 24.2	2, 6 and 12 months	**↔** FeNO **↔** Suptum eosinophils, lymphocytes, macrophages, and neutrophils **↔** Sputum IL-1RA, IL-2, IL-2R, IL-4, IL-6, IL-10, IL-13, IL-15, G-CSF, GM-CSF, IFN-γ, CCL2, CCL4, CXCX-8, CXCL-10, EGF, VEGF
**Ramos-Martínez et al.** (35)**, 2018**	RCT, Mexico	86 (18-50 years old, male = 12.8%, not specified)	Not specified	Not specified	Not specified	Not specified	Daily (6 months)	**Intervention:** vitamin D (calcitriol) (0.25 μg/day) **+** β2 agonists + ICS (n = 43) **Control:** Placebo **+** β2 agonists + ICS (n = 43)	Not specified	Not specified	6 months	**↑**Serum IL-10 and IFN- γ, sputum LL-37 **↓** Serum IL-5, IL-9, IL-13 and IgE, blood eosinophils
**Rosser et al.** (36)**, 2022**	RCT, USA	174 (6-16 years old, male = 60.3%, not specified)	Not specified	Not specified	**Z- scores:** 0.92 ± 1.07	**Z- scores:** 0.92 ± 1.26	Daily (not specified)	**Intervention:** vitamin D (cholecalciferol) (4,000 IU/day) **+** Fluticasone (n = 84) **Control:** Placebo **+** Fluticasone (n = 90)	56.4 ± 11.5	57.4 ± 11.5	48 weeks	**↔** Serum IgE
**Shabana et al.** (37)**, 2019**	RCT, Egypt	79 (> 19, 34.7 ± 7.2 years old, male = 64.5%, Mild: 21 (26.6%) - Moderate: 19 (24.1%) - Severe: 39 (49.4%))	5.03 ± 1.90	5.90 ± 2.50	25.15 ± 5.75	26.68 ± 2.82	Single bolus	**Intervention:** vitamin D (cholecalciferol) (300,000 IU) (n = 42) **Control:** Placebo (n = 37)	43.8 ± 6.8	45.3 ± 7.2	3 months	**↑**Serum IL-10 **↓**Serum IL-17A and IL17A/IL10 ratio
**Tachimoto et al.** (38)**, 2016**	RCT, Japan	89 (6-15 years old, Male = 56.2%, not specified)	Not specified	Not specified	17.6 ± 2.6	17.4 ± 2.9	Daily (2 months)	**Intervention:** vitamin D (cholecalciferol) (800 IU/day) (n = 54) **Control:** placebo (n = 35)	71.2 (57.4, 82.4)	72.4 (62.4, 87.4)	2 and 6 months	**↔** Serum IgE
**Thakur et al.** (39)**, 2021**	RCT, India	60 (6-11 years old, male = 56.7%, moderate persistent asthma)	Not specified	Not specified	**Z- scores:** −0.90 (−1.5 to −0.4)	**Z-scores:** −0.83 (−1.4 to −0.3)	Daily (3 months)	**Intervention:** vitamin D (cholecalciferol) (2,000 IU/day) (n = 30) **Control:** Placebo (n = 30)	39.4 ± 20.5	41.2 ± 24.7	3 months	**↔** FeNO

25(OH)D, 25-hydroxyvitamin D; IU, international unit; N, number; UK, United Kingdom; USA, United States of America; EBC, Exhaled breath condensate; BMI, Body mass index. Values are reported as mean ± standard deviation, median (first interquartile, third interquartile) or median (minimum - maximum), ↑, statistically significant increase or higher value in the intervention vs. control groups; ↓, statistically significant decrease or lower value in the intervention vs. control groups; ↔, no statistically significant effect.

* CCL2, Chemokine (C-C motif) ligand 2; CCL4, Chemokine (C-C motif) ligand 4; CXCL10, Chemokine (C-X-C motif) ligand 10; CXCL8, Chemokine (C-X-C motif) ligand 8; CXCL-10, Chemokine (C-X-C motif) ligand 10; ECP, Eosinophil cationic protein; EGF, Epidermal growth factor; FeNO, Fractional exhaled nitric oxide; G-CSF, Granulocyte-colony stimulating factor; GM-CSF, Granulocyte-macrophage colony-stimulating factor; HsCRP, High-sensitivity C-reactive protein; IFN-γ, Interferon gamma; IL-1RA, Interleukin 1 receptor antagonist; IL-2, Interleukin 2; IL-2R: Interleukin 2 receptor, IL-4, Interleukin 4; IL-5, Interleukin 5; IL-6, Interleukin 6; IL-9, Interleukin 9; IL-10, Interleukin 10; IL-13, Interleukin 13; IL-15: Interleukin 15; IL-17, Interleukin 17; IL-17A, Interleukin 17A; IgA, Immunoglobulin A; IgE, Immunoglobulin E; LL-37, Cathelicidin antimicrobial peptide; TNF-α, Tumor necrosis factor alpha; VEGF, Vascular endothelial growth factor.

### 3.3 Risk of bias

Of the thirteen RCTs, nine were assessed as low risk of bias (28–32, 34, 35, 38, 39) specifically describing appropriate randomization methods, complete reporting of outcomes and objective measurement of outcomes (**Table 2**). Four RCTs were rated as having unclear risk of bias (27, 33, 36, 37), with methodological quality limited by group imbalance in baseline patients regarding sex (27, 36, 38), baseline 25(OH)D levels (27, 32, 33), and disease duration (27), as well as bias in the selective reported outcomes. All studies included in our review demonstrated an equal distribution of age and BMI, across both intervention groups.

**Table 2:** Risk of bias summary based on Cochrane Systematic Review Guidelines for included randomized controlled trials.

Study	Domain 1	Domain 2	Domain 3	Domain 4	Domain 5	Overall risk of bias
**Abbas et al.** (27)	Some concerns	Some concerns	Low	Low	Some concerns	Unclear
**Andújar-Espinosa et al.** (28)	Low	Low	Low	Low	Low	Low
**BarYoseph et al.** (29)	Low	Low	Low	Low	Some concerns	Low
**Castro et al.** (30)	Low	Low	Low	Low	Low	Low
**De Groot et al.** (31)	Low	Low	Low	Low	Some concerns	Low
**Dodamani et al.** (32)	Low	Low	Low	Low	Low	Low
**Kerley et al.** (33)	Some concerns	Some concerns	Low	Low	Some concerns	Unclear
**Martineau et al.** (34)	Low	Low	Low	Low	Low	Low
**Ramos-Martínez et al.** (35)	Some concerns	Low	Low	Low	Some concerns	Low
**Rosser et al.** (36)	Some concerns	Some concerns	Low	Low	Some concerns	Unclear
**Shabana et al.** (37)	Some concerns	Some concerns	Low	Low	Some concerns	Unclear
**Tachimoto et al.** (38)	Some concerns	Low	Low	Low	Some concerns	Low
**Thakur et al.** (39)	Low	Low	Low	Low	Some concerns	Low

Domain1: Bias arising from the randomization process; Domain 2: Bias due to deviations from intended interventions; Domain 3: Bias due to missing outcome data; Domain 4: Bias in measurement of the outcome, Domain 5: Bias in selection of the reported result.

### 3.4 Co-primary outcomes

#### 3.4.1 IgE

**Figure 2:**
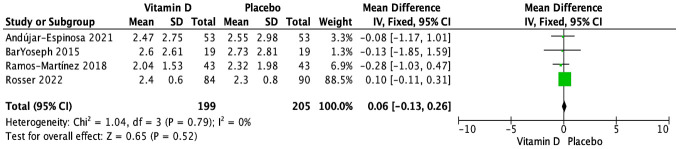
Forest plot of randomized controlled trials investigating the effects of vitamin D supplementation on serum total IgE (IU/mL) measured at the end of 6- to 48-week trials. Mean group differences in a log scale are presented with 95% CIs and calculated with the fixed-effects model. Heterogeneity was quantified by I^2^ at a significance of P < 0.10.

While eight studies assessed the impact of vitamin D supplementation on serum total IgE (IU/mL) (28, 29, 31–33, 35, 36, 38), only four trials could be aggregated (28, 29, 35, 36); three RCTs tested doses of cholecalciferol ranging from 14,000 to 16,000 IU (28, 29, 36), and one trial tested a dose of 0.25 μg of calcitriol (35). Overall, there was no statistically significant effect of vitamin D supplementation on serum IgE when measured at the endpoint, which ranged from 6 to 48 weeks (N = 404 subjects; MD [95% CI]: 0.06 [-0.13, 0.26] IU/mL; P = 0.52; I^2^ = 0%) (**Figure 2**). Furthermore, there were no statistically significant subgroup differences based on age group (children vs. adults) (**Supplementary Figure S1**), baseline vitamin D status (25(OH)D ≥50 nmol/L vs. 25(OH)D <50 nmol/L vs not specified) (**Supplementary Figure S2**), dose regimen (daily vs. weekly) (**Supplementary Figure S3**), and vitamin D form (cholecalciferol vs. calcidiol vs. calcitriol) (**Supplementary Figure S4**). The sensitivity analysis, which included only three RCTs at a low risk of bias (28, 29, 35), revealed no significant impact of vitamin D on serum total IgE (N = 230 subjects; MD [95% CI]: -0.21 [-0.79, 0.37] IU/mL; P = 0.49) (**Supplementary Figure S5**).

The other four RCTs were not included in the meta-analysis for various reasons. Two trials reported results narratively without providing quantitative data (33, 38); one trial found no significant effect on serum IgE levels at 15 weeks in children receiving daily supplementation of 2,000 IU of vitamin D (33), while the other reported no significant effect at both 2 and 6 months in children supplemented with daily doses of 800 IU of vitamin D (38). The remaining two trials reported quantitative data in a way that could not be aggregated (31, 32). One RCT, involving both adults and children given a 400,000 IU vitamin D or placebo bolus, showed no significant effect at 9 weeks. However, due to skewed data, the median (IQR) could not be converted into mean (SD) (vitamin D: 29 (13, 88); placebo: 47 (4, 264)) (31). Another RCT reported the percentage change from baseline in IgE levels, indicating no statistically significant difference at 6 months in children and adults taking a weekly supplementation of 60,000 IU of vitamin D (vitamin D: 8.6 (-13.4 to 30.6)%; placebo: 9.9 (-8.3 to 28.1)%) (32).

#### 3.4.2 Blood eosinophils

**Figure 3:**
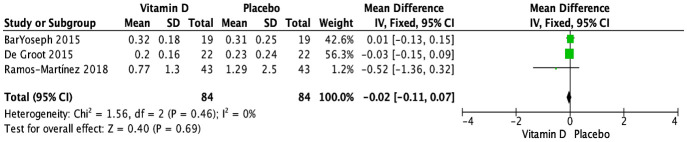
Forest plot of randomized controlled trials investigating the effects of vitamin D supplementation on blood eosinophils (10^3^/μL) measured at the end of 6-week to 6-month trials. Values are mean differences with 95% CIs determined with the use of fixed-effects model. Heterogeneity was quantified by I^2^ at a significance of P < 0.10.

The impact of vitamin D supplementation on blood eosinophils (10^3^/μL) was evaluated in four trials (29, 31, 33, 35). One RCT narratively mentioned no significant effect at 15 weeks in children receiving daily supplementation of 2,000 IU of vitamin D (33). Three RCTs provided sufficient data that could be pooled (29, 31, 35); they tested doses ranging from 14,000 to 400,000 IU of cholecalciferol, as well as a dose of 0.25 μg of calcitriol. Overall, no significant effect of vitamin D supplementation was observed on blood eosinophils measured at the end of the study periods, which ranged from 6 weeks to 6 months (MD [95% CI]: -0.02 [-0.11, 0.07] 10^3^/μL; P = 0.69; I^2^ = 0%) (**Figure 3**). Similarly, there were no significant subgroup differences based on age group (**Supplementary Figure S6**) or vitamin D form (**Supplementary Figure S7**). Due to varying administration frequencies in the three studies (daily, weekly, single bolus) and insufficient reporting of baseline serum 25(OH)D levels [≥50 nmol/L in two studies (29, 31) and not specified in one trial (35)], subgroup analyses for baseline 25(OH)D level and administration frequency could not be conducted. Given that all studies exhibited a low risk of bias, no sensitivity analysis was conducted based on study quality. The exclusion of studies with initial results reported as median (IQR) did not alter the overall findings, with no significant impact of vitamin D supplementation compared to placebo (n = 2 RCTs (29, 35); MD [95% CI]: -0.17 [-0.52, 0.19] 10^3^/μL; P = 0.36) (**Supplementary Figure S8**).

### 3.5 Secondary outcomes

#### 3.5.1 Sputum eosinophils

Three RCTs assessed the impact of vitamin D supplementation on sputum eosinophils (%). These studies reported no significant effect at endpoints ranging from 9 weeks to 12 months (30, 31, 34), but their data could not be pooled. One RCT, which narratively reported results in adults after administering a single bolus of 100,000 IU of vitamin D followed by a daily dose of 4,000 IU, did not provide numeric data (30). The other two RCTs, both conducted in adults and children, reported skewed data, making the transformation of medians (IQR) to means (SD) unfeasible. One trial tested bi-monthly supplementation with 120,000 IU (vitamin D: 1.25 (0.42-5.25) %; placebo: 3.80 (0.42-5.42) %) (34), while the other tested a single bolus of 400,000 IU (vitamin D: 0.7 (0.2-11.4) %; placebo: 3.9 (0.2-50.9) %) (31).

#### 3.5.2 FeNO

**Figure 4:**
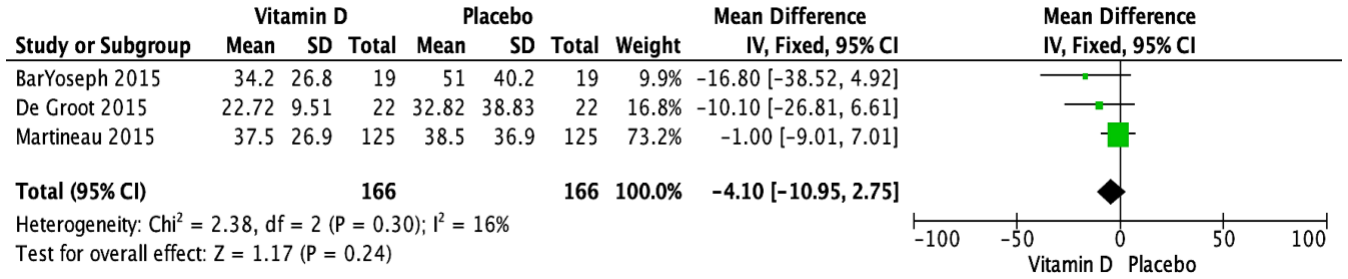
Forest plot of randomized controlled trials investigating the effects of vitamin D supplementation on FeNo (ppb) measured at the end of the study at 6 weeks to 12 months. Values are mean differences with 95% CIs determined with the use of fixed-effects model. Heterogeneity was quantified by I^2^ at a significance of P < 0.10.

The impact of vitamin D supplementation on FeNO was examined in four trials (29, 31, 34, 39). A pooled meta-analysis of three RCTs (29, 31, 34) revealed no significant effect at the endpoint, with doses ranging from 14,000 to 400,000 IU over periods from 6 weeks to 12 months (MD [95% CI]: -4.10 [-10.95, 2.75] ppb; P = 0.24; I^2^ = 16%) (**Figure 4**). There were no significant subgroup differences based on baseline vitamin D status (**Supplementary Figure S9**) or dose regimen (**Supplementary Figure S10**). Since all the studies had varied patient populations (children, adults, or both) and used the same form of vitamin D (cholecalciferol), these subgroup analyses could not be conducted. The RCT not included in the meta-analysis reported skewed data, preventing conversion of medians (IQR) to means (SD); it reported no significant effect at 3 months in children receiving daily supplementation of 2,000 IU of vitamin D (vitamin D: 16 (10-24.5) ppb; placebo: 10 (8.3-21.8) ppb) (39). As all the studies demonstrated a low risk of bias, no further sensitivity analyses were conducted based on study quality.

#### 3.5.3 IL-10

**Figure 5:**
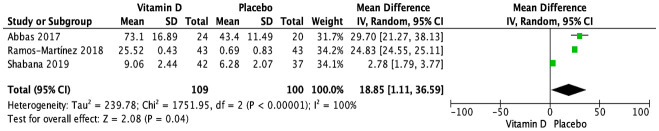
Forest plot of randomized controlled trials investigating the effects of vitamin D supplementation on serum IL-10 (pg/mL) measured at the end of the study at 3 to 6 months. Values are mean differences with 95% CIs determined with the use of random-effects models. Heterogeneity was quantified by I^2^ at a significance of P < 0.10.

Five trials investigated the impact of vitamin D supplementation on serum IL-10 (pg/mL) (27, 32, 33, 35, 37). Three RCTs contributing data for pooled analysis (27, 35, 37) demonstrated a significantly higher serum IL-10 measured at the endpoint in the vitamin D supplementation group compared to placebo (MD [95% CI]: 18.85 [1.11, 36.59] pg/mL; P = 0.04; I^2^ = 100%) (**Figure 5**). These results were observed at 3 to 6 months in patients who received single vitamin D doses ranging from 2,000 to 300,000 IU or daily doses (0.25 ug) of calcitriol. No significant subgroup differences were noted based on age group (**Supplementary Figure S11**) or the form of vitamin D (**Supplementary Figure S12**). As all studies varied in the frequency of administration (single or daily doses, and single bolus) and baseline serum 25(OH)D levels (<25 nmol/L vs. 25-50 nmol/L vs. not specified), subgroup analyses could not be conducted. The two RCTs not included in the meta-analysis reported no significant effect: one RCT, conducted among adults and children with a weekly supplementation of 60,000 IU of vitamin D, had skewed results, reporting medians (IQRs) at 6 months [vitamin D: 7.9 (4.3, 31.8); placebo: 11 (1, 31.8)] (32). Another RCT, which focused on children supplemented daily with 2,000 IU of vitamin D, provided results as a median change from baseline (IQR) at 15 weeks [vitamin D: -13 (-25 to 3); placebo: -17 (-26 to 27)] (33). Given that two studies had unclear risks of bias and one study exhibited a low risk of bias, conducting a sensitivity analysis based on study quality was not feasible.

As for IL-10 levels in other bodily fluids, one trial reported no significant effect of vitamin D supplementation on sputum IL-10 (pg/mL) at 12 months in children and adults with bi-monthly supplementation of 120,000 IU of vitamin D [median (IQR) for vitamin D: 0.0 (0.0 - 2.2); Placebo: 1.6 (0.0 - 4.6)] (34). Another trial showed no significant effect on exhaled breath condensate IL-10 (pg/mL) at 6 weeks post-intervention in children taking a 14,000 IU weekly supplementation of vitamin D (mean ± SD for vitamin D: 1.27 ± 0.45; placebo: 1.53 ± 0.53) (29).

#### 3.5.4 Other inflammatory biomarkers

RCTs have explored the impact of vitamin D supplementation on various other biomarkers, namely: (i) pro-inflammatory biomarkers of type 2 inflammation, including eosinophil cationic protein (ECP) (33), IL-4 (29, 32, 34), IL-5 (29, 35), and IL-13 (34, 35); (ii) pro-inflammatory biomarkers of non-type 2 inflammation such as IL-2 (34), IL-6 (32, 34), IL-9 (35), IL-15 (34), IL-17 (29, 32, 37), IFN-γ (29, 34, 35), TNF-α (27), HsCRP (29, 33), neutrophils (31, 34), granulocyte-colony stimulating factor (G-CSF), granulocyte-macrophage colony-stimulating factor (GM-CSF), chemokine (C-C motif) ligand (CCL)-2, CCL4, chemokine (C-X-C motif) ligand (CXCL)-8, CXCL-10, epidermal growth factor (EGF) and VEGF (34); (iii) anti-inflammatory biomarkers including IL-1RA, IL-2R (34), and cathelicidin LL-37 (33, 35); and (iv) non-specific biomarkers such as lymphocytes and macrophages (34), IgA (33), and IL-17A/IL-10 ratio (37). However, the results could not be aggregated due to the heterogeneity of the biological fluid used for measurements, the inability to convert medians and IQRs into means and SDs due to skewed data distribution, or the insufficient number of studies (fewer than 2 studies per biomarker) necessary to perform a meta-analysis.

## 4 Discussion

This systematic review and meta-analysis suggest that vitamin D supplementation may not have a statistically significant impact on pro-inflammatory biomarkers of type 2 inflammation in children and adults with asthma, specifically on serum IgE, blood eosinophils, and FeNO, when measured at endpoints ranging from 6 weeks to 12 months post-randomization. However, the analysis indicated that serum IL-10 levels, an anti-inflammatory biomarker, was higher in vitamin D supplemented groups compared to placebo groups at the end of studies lasting 3 to 6 months in three trials. Due to poor or inconsistent reporting, we were not able to pool changes from baseline in any biomarkers, a within-patient metric that could have yielded more precise data. The narrative synthesis also suggested no significant impact of vitamin D supplementation on sputum eosinophils (3 RCTs), sputum IL-10 (1 RCT), and exhaled breath condensate IL-10 (1 RCT) at endpoints. The impact of vitamin D on other inflammatory biomarkers was less clear due to the limited number of studies that reported these data in a way that permitted aggregation.

As for type 2 pro-inflammatory biomarkers, we did not observe a statistically significant impact at the endpoint of supplementation in patients with asthma who were administered various vitamin D regimens with diverse frequencies, ranging from daily doses (4,000 IU), weekly doses (14,000 IU), bi-monthly doses (120,000 IU), to larger bolus amounts (400,000 IU) using cholecalciferol. Some participants also received weekly doses of 16,000 IU of calcidiol or daily doses of calcitriol (0.25μg). Our results align with previous findings. A recent systematic review and meta-analysis of three RCTs (28, 35, 36) by Williamson et al. found that vitamin D supplementation did not significantly affect serum IgE at the endpoint in children and adults with asthma (5). Although they excluded a study because of its shorter duration of follow-up (6 weeks), which we incorporated into our review (29), the conclusion on serum IgE was consistent with our reports. Notably, their review accessed unpublished data, allowing them to also aggregate results on sputum eosinophils at the endpoint (30, 34, 38), where no significant impact was observed for children and adults with asthma (5). Another meta-analysis on blood eosinophils from three RCTs involving children and adults (18) incorporated the same studies as ours (29, 31, 35) and similarly concluded that vitamin D supplementation had no statistically significant effect. As for FeNO, our findings, based on 3 RCTs (29, 31, 34), are in line with two previous meta-analyses (19, 20) that considered only two RCTs from our selection and also examined biomarker values at the endpoint. Of note, our results align with a recent review by Visser et al. (21), which synthesized the current evidence on the effects of several dietary interventions, including vitamin D supplementation on type 2 pro-inflammatory biomarkers in adolescents (≥ 12 years old) and adults. This review included only five studies from our selection of 13 RCTs (30–32, 34, 35), along with an additional two pre-post studies (40, 41). Although unable to conduct a meta-analysis, this review narratively reported that most of the included studies observed non-statistically significant decreases in one or more markers of type 2 inflammation, such as sputum and blood eosinophils, as well as FeNO. However, three studies showed a statistically significant decrease in eosinophils. In our review exclusively considering RCTs to significantly reduces the risk of bias and confounding variables, thus providing stronger evidence for cause-and-effect relationships, vitamin D supplementation in various dosages over periods ranging from 6 weeks to 12 months showed no evidence of an effect on serum IgE and blood eosinophils, and it had neither a statistically significant nor clinically important effect on FeNO at the endpoint. The evidence is derived from three studies at low risk of bias for each biomarker.

As for anti-inflammatory biomarkers, various dosing regimens of vitamin D supplementation, ranging from single administration of 2,000 to 300,000 IU or daily doses of calcitriol, were associated with significantly higher IL-10 levels at the endpoint compared to placebo (3 RCTs). This finding contrasts with a prior systematic review of 4 RCTs by Wang et al. (18), which reported no significant effect on IL-10 levels at the endpoint (18). When comparing included studies, the Wang et al. (18) review incorporated two of the 3 studies that were a part of our quantitative analysis (35, 37) and two others that we included only in our narrative review (29, 32). We excluded the latter two from quantitative analysis either because of data asymmetry that made the transformation from median to mean invalid (32) or because IL-10 measurement in a different biological compartment than serum (i.e., exhaled breath condensate) (29). Moreover, we included two additional studies that they omitted from their analysis (27, 34). Our findings also differ from another Wang et al. (19) IL-10 meta-analysis which reported no effect at endpoint (19). The Wang et al. (19) review had included the same three studies we did, plus an additional *ex vivo* trial (42) in which IL-10 was measured in cell cultures of peripheral blood mononuclear cells that were treated with the Derp2 allergen at a concentration of 10 mg/mL for 72 hours, a trial we chose to exclude because of the ex-vivo allergen stimulation.

Yet, the higher IL-10 observed at the endpoint in the vitamin D supplemented group is in line with several preclinical and clinical studies showing that vitamin D may increase IL-10 (9). This anti-inflammatory cytokine can be expressed by various cell types, including dendritic cells, monocytes, and T and B lymphocytes (43). Indeed, vitamin D enhances IL-10 production by exerting an inhibitory effect on dendritic cell maturation and promoting the development of suppressive IL-10-producing Treg cells (44). IL-10, in turn, can regulate and modulate the expression of numerous immune cells and pro-inflammatory cytokines (45). Collectively, our findings suggest an anti-inflammatory impact of vitamin D supplementation *in vivo*, as previously observed in *ex vivo.* Further investigation of the effect of vitamin D supplementation on IL-10 in asthma patients is indicated.

Analogous to previous reviews, we were unable to aggregate data for other pro-inflammatory biomarkers related to type-2 or non-type 2 inflammation, as well as non-specific biomarkers whose pro- or anti-inflammatory roles are unclear or variable. This was mainly due to the small number of studies for each biomarker (fewer than two studies) and the differences in the biological samples analyzed. Only one prior Wang et al. (18) review managed to aggregate data on other inflammatory biomarkers such as IL-5 for patients with asthma at endpoints and found no significant impact (18). They included two studies that we also included in our review but had elected not to aggregate their data since they come from two different biological compartments (serum and exhaled breath condensate) (29, 35). Indeed, our review highlighted conflicting results between different biological samples for the same cytokine, hence the importance of separating them for rigorous analysis.

### 4.1 Strengths and limitations

Our systematic review and meta-analysis have several strengths. Firstly, our study is the most recent, encompassing additional trials not included in previous systematic reviews and meta-analyses. Secondly, our study was wide-ranging, as we incorporated a diverse array of inflammatory biomarkers into our quantitative and qualitative assessments. These biomarkers were measured *in vivo*, contrasting with many studies that explored the mechanism of action of vitamin D either *ex vivo* or *in vitro*. Lastly, we demonstrate our methodological rigor by excluding certain studies due to concerns like invalid data conversion (from median to mean) when the symmetrical distribution assumption was not met, measurements from distinct biological compartments such as sputum, serum, and exhaled breath condensate, and/or *ex-vivo* experimental designs that previous meta-analyses had included.

We recognized several limitations, mostly due to the paucity of available data. First, although we incorporated evidence from 13 RCTs assessing the effect of vitamin D supplementation on at least one inflammatory biomarker, the variability in biomarkers measured only permitted the aggregation of data from no more than four trials for IgE meta-analysis and three trials each for blood eosinophils, FeNO, and IL-10 meta-analyses. Second, given the small number of trials, we were unable to adjust for, or explore, different measurement time points for the inflammatory biomarkers and variations in vitamin D supplementation modalities and posologies across trials. We recognize that the timing of biomarkers measurements in relation to the onset and type of vitamin D supplementation could be important in discerning between immediate, delayed, temporary, or sustained effects. Third, the included studies did not report dietary recommendations to maximize absorption of vitamin D supplements, or status of other vitamin, such as vitamin A. Fourth, there was noticeable heterogeneity in population (including both children and adults), intervention (using different forms, dosing regimens, and durations of vitamin D supplementation) and inflammatory biomarkers considered. Despite subgroup analyses, we couldn’t account for this heterogeneity for IL-10 meta-analysis. Additionally, our research was impeded by incomplete reporting or inaccessible individual data, which restricted our ability to aggregate all relevant evidence for the meta-analysis. Finally, it is uncertain whether the effect of vitamin D on inflammation biomarkers is direct or if it occurs indirectly through other pathways; future research should utilize causal inference methods that can distinguish between the direct and indirect effects of vitamin D on inflammatory biomarkers.

## 5 Conclusion

In conclusion, this systematic review and meta-analysis suggests that vitamin D supplementation does not significantly impact key type-2 inflammatory biomarkers (serum IgE, blood and sputum eosinophils, and FeNO) in individuals with asthma. However, it appears to increase levels of the anti-inflammatory biomarker, IL-10, suggesting that vitamin D supplementation may exert an anti-inflammatory effect in asthma. Future research and investigators should consider sharing individual patient data, including changes from baseline values, to enable data aggregation for subsequent meta-analyses and consider causal inference methods to explore direct and indirect effects of vitamin D on inflammation biomarkers.

## Data availability statement

The original contributions presented in the study are included in the article/**Supplementary Material**. Further inquiries can be directed to the corresponding author.

## Author contributions

AEA: Conceptualization, Methodology, Data curation, Formal analysis, Writing – original draft. HD: Validation, Writing – review & editing. PD: Investigation, Writing – review & editing. HT: Conceptualization, Methodology, Supervision, Writing – review & editing. FMD: Conceptualization, Methodology, Supervision, Writing – review & editing.
