# Supplementary material for: The effects of vitamin D supplementation on inflammatory biomarkers in patients with asthma: a systematic review and meta-analysis of randomized controlled trials
Source: Front Immunol. 2024 Mar 13;15:1335968. doi: 10.3389/fimmu.2024.1335968 (PMC10965564; doi:10.3389/fimmu.2024.1335968)
Supplement: Supplementary file 1 [file DataSheet_1.docx]

Supplementary Material

# Supplementary methods

**Method S1**. Search strategy

## PubMed

| #1 | Vitamine D | Vitamin D[mh] OR Vitamin D Deficiency[MH:noexp] OR "25OHD*"[tiab] OR "25OH-D*"[tiab] OR "25-OHD*"[tiab] OR "25-OH-D*"[tiab] OR "dht intensol"[tiab] OR "Dihydroxyvitamin D*"[tiab] OR "Dihydroxyvitamins D*"[tiab] OR "dnt intensol"[tiab] OR "Hydroxyvitamin D*"[tiab] OR "Hydroxyvitamins D*"[tiab] OR "MC 1288"[tiab] OR "MC1288"[tiab] OR "vitamin D*"[tiab] OR "vitamins D*"[tiab] OR antitanil[tiab] OR antitetanin*[tiab] OR atecen[tiab] OR bocatriol[tiab] OR calcamin*[tiab] OR Calcidiol*[tiab] OR Calcifediol*[tiab] OR Calciferol*[tiab] OR calcijex[tiab] OR calcinosefaktor[tiab] OR Calciol*[tiab] OR calcitriol[tiab] OR Calderol*[tiab] OR Cholercalciferol*[tiab] OR decostriol[tiab] OR Dedrogyl*[tiab] OR dichistrolum[tiab] OR dichysterol[tiab] OR dichystrol[tiab] OR dihydral[tiab] OR dihydrotachysterin*[tiab] OR Dihydrotachysterol*[tiab] OR dihydroxycholecalciferol*[tiab] OR DihydroxyvitaminD*[tiab] OR DihydroxyvitaminsD*[tiab] OR dikystrol[tiab] OR dygratyl[tiab] OR Ercalcidiol*[tiab] OR Ergocalciferol*[tiab] OR Hidroferol*[tiab] OR Hydroxycalciferol*[tiab] OR Hydroxycholecalciferol*[tiab] OR Hydroxyergocalciferol*[tiab] OR HydroxyvitaminD*[tiab] OR HydroxyvitaminsD*[tiab] OR hytakerol[tiab] OR manipal[tiab] OR osteotriol[tiab] OR parterol[tiab] OR renatriol [tiab] OR silkis[tiab] OR sitriol[tiab] OR Soltriol[tiab] OR tachidon[tiab] OR tachystin*[tiab] OR tachystol[tiab] OR tetilan[tiab] OR tirocal[tiab] OR vitaminD*[tiab] OR vitaminsD*[tiab] |
| --- | --- | --- |
| #2 | Asthme | Asthma[mh] OR Asthma*[tiab] |
| #3 | Biomarqueurs d'inflammation | Biomarkers[mh:noexp] OR Inflammation[mh:noexp] OR "Immunoglobulin E"[Mh:noexp] OR "C-Reactive Protein"[mh] OR Calcitonin[mh] OR Procalcitonin[mh] OR "Cell Count"[mh] OR "Blood Cells"[mh] OR "Fractional Exhaled Nitric Oxide Testing"[mh] OR Hematocrit[mh] OR Cytokines[mh] OR Hemoglobins[mh:noexp] OR "Blood Sedimentation"[mh] OR "Leukocyte L1 Antigen Complex"[Mesh] OR Ferritins[mh] OR Fibrinogen[mh] OR "Serum Amyloid A Protein"[Mh] OR "8-Hydroxy-2'-Deoxyguanosine"[Mesh] OR "Cyclooxygenase 2"[Mh] OR "Receptors, Urokinase Plasminogen Activator"[mh] OR "Mast Cells"[mh] OR Macrophages[mh:noexp] OR "Acute-Phase Proteins"[mh] OR Interleukins[mh] OR "Tumor Necrosis Factors"[Mh] OR "E-Selectin"[mh] OR "Intercellular Adhesion Molecule-1"[mh] OR "Vascular Cell Adhesion Molecule-1"[mh] OR Interferons[mh] OR Dendritic Cells[mh] OR (Immunity, Innate[mh] AND Lymphocytes[mh]) OR "Killer Cells, Natural"[mh] OR "T-Lymphocytes"[mh] OR "B-Lymphocytes"[mh] OR "Antigen-Presenting Cells"[mh] OR "fibrin fragment D"[Supplementary Concept] OR "27E10 Antigen"[tiab] OR "4-1BB Ligand"[tiab] OR "4-1BBL"[tiab] OR "45S RNP"[tiab] OR "A Proliferation Inducing Ligand Protein*"[tiab] OR "Acid alpha 1-Glycoprotein*"[tiab] OR "Acidic Pancreatic Trypsin Inhibitor*"[tiab] OR "alpha 1 Antiprotease"[tiab] OR "alpha 1 Antitrypsin"[tiab] OR "alpha 1-Acid Glycoprotein*"[tiab] OR "alpha 1-Antichymotrypsin*"[tiab] OR "alpha 1-Antiproteinase"[tiab] OR "alpha 1-Glycoprotein Acid*"[tiab] OR "alpha 1-Macroglobulin*"[tiab] OR "alpha 1-Protease Inhibitor"[tiab] OR "alpha 1-Proteinase Inhibitor"[tiab] OR "alpha 2-Globulin*"[tiab] OR "alpha 2-Macroglobulin*"[tiab] OR "alpha Macroglobulin*"[tiab] OR "alpha-Macrofetoprotein*"[tiab] OR "Amyloid A Precursor"[tiab] OR "Amyloid A Protein"[tiab] OR "Amyloid A"[tiab] OR "Amyloid Fibril Protein AA"[tiab] OR "Amyloid P Component*"[tiab] OR "Amyloid Protein AA"[tiab] OR "Amyloid Protein AA"[tiab] OR "Amyloid Protein SAA"[tiab] OR "Amyloid Serum Protein SAA"[tiab] OR "Antigen L1"[tiab] OR "Antigen-Presenting Cell*"[tiab] OR "Apo 2 Ligand"[tiab] OR "Apo 3 Ligand"[tiab] OR "Apo2 Ligand"[tiab] OR "Apo3 Ligand"[tiab] OR "APRIL Protein*"[tiab] OR "B cell*"[tiab] OR "B Lymphocyte Stimulator*"[tiab] OR "B1 Cell*"[tiab] OR "B-1 Cell*"[tiab] OR "B1 Lymphocyte*"[tiab] OR "B-1 Lymphocyte*"[tiab] OR "B2 Cell*"[tiab] OR "B-2 Cell*"[tiab] OR "B2 Lymphocyte*"[tiab] OR "B-2 Lymphocyte*"[tiab] OR "B-Cell Activating Factor*"[tiab] OR "Be1 Cell*"[tiab] OR "Be2 Cell*"[tiab] OR "beta-1 Metal-Binding Globulin"[tiab] OR "beta-1B-glycoprotein"[tiab] OR "B-Lymphocyte Activating Factor*"[tiab] OR "B-Lymphocyte*"[tiab] OR "B-Lymphoid*"[tiab] OR "C3 Complement*"[tiab] OR "C3 Precursor*"[tiab] OR "Caeruloplasmin*"[tiab] OR "CD 4"[tiab] OR "CD134 Ligand"[tiab] OR "CD137 Ligand"[tiab] OR "CD153 Antigen*"[tiab] OR "CD154 Antigen*"[tiab] OR "CD178 Antigen*"[tiab] OR "CD253 Antigen*"[tiab] OR "CD254 Antigen*"[tiab] OR "CD257 Antigen*"[tiab] OR "CD30 Ligand"[tiab] OR "CD40 Ligand"[tiab] OR "CD4-Positive Lymphocyte*"[tiab] OR "CD54 Antigen*"[tiab] OR "CD70 Antigen*"[tiab] OR "CD8 Positive Lymphocyte*"[tiab] OR "CD95 Ligand"[tiab] OR "Complement 3"[tiab] OR "Complement C3"[tiab] OR "Complement Component 3"[tiab] OR "COX 2"[tiab] OR "D dimer"[tiab] OR "D1 dimer"[tiab] OR "E Selectin*"[tiab] OR "ELAM*"[tiab] OR "Endothelial Leukocyte Adhesion Molecule-1"[tiab] OR "Fas Ligand"[tiab] OR "fibrin fragment D"[tiab] OR "Herpesvirus Entry Mediator-Ligand Protein*"[tiab] OR "ICAM*"[tiab] OR "Immunoglobulin E"[tiab] OR "Immunologic Accessory Cell*"[tiab] OR "Intercellular Adhesion Molecule*"[tiab] OR "Kazal Pancreatic Trypsin Inhibitor*"[tiab] OR "L1 Antigen"[tiab] OR "LAK Cell*"[tiab] OR "LECAM-2"[tiab] OR "LIGHT Protein*"[tiab] OR "Lipocalin-2"[tiab] OR "Lymphokine Activated Killer Cell*"[tiab] OR "Neutrophil Gelatinase-Associated Lipocalin"[tiab] OR "NGAL Protein*"[tiab] OR "Oncogene 24p3 Protein*"[tiab] OR "Osteoclast Differentiation Factor*"[tiab] OR "OX40 Ligand"[tiab] OR "Pancreatic Secretory Trypsin Inhibitor*"[tiab] OR "Pro-C3"[tiab] OR "Receptor Activator of Nuclear Factor-kappa*"[tiab] OR "Serpin A3"[tiab] OR "Serum A Related Protein"[tiab] OR "Serum Amyloid A"[tiab] OR "Serum Amyloid P"[tiab] OR "Serum Amyloid Protein A"[tiab] OR "Serum Sialomucin*"[tiab] OR "Siderocalin Protein*"[tiab] OR "T cell*"[tiab] OR "T Helper"[tiab] OR "T Lymphocyte*"[tiab] OR "T4 cell*"[tiab] OR "T4 Lymphocyte*"[tiab] OR "T8 Cell*"[tiab] OR "T8 Lymphocyte*"[tiab] OR "TALL-1 Protein*"[tiab] OR "TALL2 Protein*"[tiab] OR "TALL-2 Protein*"[tiab] OR "TC1 Cell*"[tiab] OR "TC2 Cell*"[tiab] OR "Tfh Cell*"[tiab] OR "Th1 Cell*"[tiab] OR "Th17 Cell*"[tiab] OR "Th2 Cell*"[tiab] OR "Th3 Cell*"[tiab] OR "TL1 Ligand"[tiab] OR "TL1A Ligand"[tiab] OR "T-Lymphoid*"[tiab] OR "TNFS*"[tiab] OR "Tr1 Cell*"[tiab] OR "TRAIL Protein*"[tiab] OR "TRANCE Protein*"[tiab] OR "Trypsin Inhibitor Kazal Pancreatic"[tiab] OR "Tumor Associated Trypsin Inhibitor*"[tiab] OR "tumor necrosis serum*"[tiab] OR "tumour necrosis serum*"[tiab] OR "Vascular Cell Adhesion Molecule*"[tiab] OR "VCAM*"[tiab] OR 8OHdG[tiab] OR 8-OH-dG[tiab] OR 8-oxodG[tiab] OR 8-oxo-dG[tiab] OR 8-oxodGuo[tiab] OR 8-oxo-dGuo[tiab] OR A1PI[tiab] OR acute phase globulin*[tiab] OR Acute Phase Glycoprotein*[tiab] OR acute phase plasma protein*[tiab] OR acute phase protein*[tiab] OR Acute Phase Reactant*[tiab] OR acute phase response protein*[tiab] OR Amyloid Protein*[tiab] OR Apo2L[tiab] OR Apo2-L[tiab] OR "Apo-2-L"[tiab] OR Apo3L[tiab] OR Apo3-L[tiab] OR "Apo-3-L"[tiab] OR Apoferritin*[tiab] OR BAFF Ligand[tiab] OR Basophil*[tiab] OR Biomarker*[tiab] OR Blood cell*[tiab] OR Blood Count*[tiab] OR Blood Number*[tiab] OR Bloodcell*[tiab] OR BLyS Protein[tiab] OR Breg Cell*[tiab] OR C Reactive Protein[tiab] OR Cachectin[tiab] OR Cachetin[tiab] OR Calcitonin*[tiab] OR Calcitrin*[tiab] OR Calgranulin*[tiab] OR Calprotectin*[tiab] OR CD134L[tiab] OR CD27 Ligand[tiab] OR CD27L[tiab] OR CD30L[tiab] OR CD4[tiab] OR CD40L[tiab] OR CD62E[tiab] OR CD95L[tiab] OR Cell Count*[tiab] OR Cell Number*[tiab] OR Ceruloplasmin*[tiab] OR Chemokine*[tiab] OR Ciba 47175*[tiab] OR Ciba47175*[tiab] OR COX2[tiab] OR CRP[tiab] OR CycloOxygenase*[tiab] OR Cyclo-Oxygenase*[tiab] OR Cytokine*[tiab] OR Dendritic Cell*[tiab] OR Deoxyguanosine*[tiab] OR Dihydrodeoxyguanosine*[tiab] OR Dihydroguanosine*[tiab] OR Ectodysplasin*[tiab] OR eNO[tiab] OR Eosinophil*[tiab] OR Eosinophil*[tiab] OR Erythrocyte*[tiab] OR ESR[tiab] OR Exhaled Nitric Oxide*[tiab] OR exhaled NO[tiab] OR Feno[tiab] OR Ferritin*[tiab] OR Ferroxidase*[tiab] OR Fibrinogen*[tiab] OR Granulocyte*[tiab] OR Haemopexin*[tiab] OR Haptoglobin*[tiab] OR Helper Cell*[tiab] OR Hematocrit*[tiab] OR Hemoglobin*[tiab] OR Hemopexin*[tiab] OR Hpx[tiab] OR hsCRP[tiab] OR HVEML[tiab] OR HVEM-L[tiab] OR Hx[tiab] OR Hydroxydeoxyguanosine*[tiab] OR IFN[tiab] OR IgE[tiab] OR Il[tiab] OR Il6[tiab] OR Inducer Cell*[tiab] OR Inflammat*[tiab] OR innate lymphoid cell*[tiab] OR Intercrine*[tiab] OR Interdigitating Cell*[tiab] OR Interferon*[tiab] OR Interleukin*[tiab] OR Intraepithelial Lymphocyte*[tiab] OR Isoferritin*[tiab] OR Isotransferrin*[tiab] OR Langerhans Cell*[tiab] OR LE Cell*[tiab] OR Leif[tiab] OR Leucocyte*[tiab] OR Leukocyte*[tiab] OR Lymphocyte*[tiab] OR Lymphotoxin*[tiab] OR Macrophage*[tiab] OR MAIT Cell*[tiab] OR Marker*[tiab] OR Mast Cell*[tiab] OR Mastocyte*[tiab] OR Monocyte*[tiab] OR Natural Killer Cell*[tiab] OR neutrophil*[tiab] OR NK Cell*[tiab] OR OPGL[tiab] OR Orosomucoid*[tiab] OR Osteoprotegerin Ligand[tiab] OR OX40L[tiab] OR Oxodeoxyguanosine*[tiab] OR Oxoguanosine[tiab] OR Pct[tiab] OR Plasma cell*[tiab] OR Plasmacyte*[tiab] OR Platelet*[tiab] OR procalcitonin*[tiab] OR Prolastin[tiab] OR RANK Ligand[tiab] OR RANKL[tiab] OR RbC[tiab] OR RbCC[tiab] OR Reticulocyte*[tiab] OR Seromucoid*[tiab] OR Serotransferrin*[tiab] OR Serpin A1[tiab] OR Siderophilin*[tiab] OR Soluble urokinase Plasminogen Activator Receptor*[tiab] OR SPINK1[tiab] OR SuPAR[tiab] OR THANK Protein[tiab] OR Thymocyte*[tiab] OR Thyrocalcitonin*[tiab] OR tissue necrosis factor*[tiab] OR TNF[tiab] OR TNFa*[tiab] OR Transferrin*[tiab] OR Treg Cell*[tiab] OR Tumor Necrosis Factor*[tiab] OR Tumour Necrosis Factor*[tiab] OR Veiled Cell*[tiab] OR WbC[tiab] OR WbCC[tiab] OR Zemaira[tiab] |
| #4 | Animaux | (Animals[mh] NOT humans[mh]) OR rat[tiab] OR rats[tiab] OR mouse[tiab] OR mice[tiab] OR murine[tiab] OR Guinea Pig*[tiab] |
| #5 | Combinaison | (#1 AND #2 AND #3 AND (english[LA] OR French[LA])) NOT #4 |

## Ovid MEDLINE(R) ALL

| 1 | Vitamine D | Exp Vitamin D/ OR Vitamin D Deficiency/ OR (25OHD* OR 25OH-D* OR 25-OHD* OR 25-OH-D* OR dht intensol OR Dihydroxyvitamin D* OR Dihydroxyvitamins D* OR dnt intensol OR Hydroxyvitamin D* OR Hydroxyvitamins D* OR MC 1288 OR MC1288 OR vitamin D* OR vitamins D* OR antitanil OR antitetanin* OR atecen OR bocatriol OR calcamin* OR Calcidiol* OR Calcifediol* OR Calciferol* OR calcijex OR calcinosefaktor OR Calciol* OR calcitriol OR Calderol* OR Cholercalciferol* OR decostriol OR Dedrogyl* OR dichistrolum OR dichysterol OR dichystrol OR dihydral OR dihydrotachysterin* OR Dihydrotachysterol* OR dihydroxycholecalciferol* OR DihydroxyvitaminD* OR DihydroxyvitaminsD* OR dikystrol OR dygratyl OR Ercalcidiol* OR Ergocalciferol* OR Hidroferol* OR Hydroxycalciferol* OR Hydroxycholecalciferol* OR Hydroxyergocalciferol* OR HydroxyvitaminD* OR HydroxyvitaminsD* OR hytakerol OR manipal OR osteotriol OR parterol OR renatriol OR silkis OR sitriol OR Soltriol OR tachidon OR tachystin* OR tachystol OR tetilan OR tirocal OR vitaminD* OR vitaminsD*).ti,ab,kw,kf |
| --- | --- | --- |
| 2 | Asthme | Exp Asthma/ OR Asthma*.ti,ab,kw,kf |
| 3 | Biomarqueurs d'inflammation | Biomarkers/ OR Inflammation/ OR Immunoglobulin E/ OR exp C-Reactive Protein/ OR exp Calcitonin/ OR Exp Procalcitonin/ OR exp Cell Count/ OR exp Blood Cells/ OR exp Fractional Exhaled Nitric Oxide Testing/ OR Exp Hematocrit/ OR Exp Cytokines/ OR Hemoglobins/ OR Exp Blood Sedimentation/ OR Exp Leukocyte L1 Antigen Complex/ OR Exp Ferritins/ OR Exp Fibrinogen/ OR Exp Serum Amyloid A Protein/ OR Exp 8-Hydroxy-2'-Deoxyguanosine/ OR Exp Cyclooxygenase 2/ OR Exp Receptors, Urokinase Plasminogen Activator/ OR Exp Mast Cells/ OR Macrophages/ OR Exp Acute-Phase Proteins/ OR Exp Interleukins/ OR Exp Tumor Necrosis Factors/ OR Exp E-Selectin/ OR Exp Intercellular Adhesion Molecule-1/ OR Exp Vascular Cell Adhesion Molecule-1/ OR Exp Interferons/ OR Exp Dendritic Cells/ OR (Exp Immunity, Innate/ AND Exp Lymphocytes/) OR Exp Killer Cells, Natural/ OR Exp T-Lymphocytes/ OR Exp B-Lymphocytes/ OR Exp Antigen-Presenting Cells/ OR (27E10 Antigen OR 4-1BB Ligand OR 4-1BBL OR 45S RNP OR A Proliferation Inducing Ligand Protein* OR Acid alpha 1-Glycoprotein* OR Acidic Pancreatic Trypsin Inhibitor* OR alpha 1 Antiprotease OR alpha 1 Antitrypsin OR alpha 1-Acid Glycoprotein* OR alpha 1-Antichymotrypsin* OR alpha 1-Antiproteinase OR alpha 1-Glycoprotein Acid* OR alpha 1-Macroglobulin* OR alpha 1-Protease Inhibitor OR alpha 1-Proteinase Inhibitor OR alpha 2-Globulin* OR alpha 2-Macroglobulin* OR alpha Macroglobulin* OR alpha-Macrofetoprotein* OR Amyloid A Precursor OR Amyloid A Protein OR Amyloid A OR Amyloid Fibril Protein AA OR Amyloid P Component* OR Amyloid Protein AA OR Amyloid Protein AA OR Amyloid Protein SAA OR Amyloid Serum Protein SAA OR Antigen L1 OR Antigen-Presenting Cell* OR Apo 2 Ligand OR Apo 3 Ligand OR Apo2 Ligand OR Apo3 Ligand OR APRIL Protein* OR B cell* OR B Lymphocyte Stimulator* OR B1 Cell* OR B-1 Cell* OR B1 Lymphocyte* OR B-1 Lymphocyte* OR B2 Cell* OR B-2 Cell* OR B2 Lymphocyte* OR B-2 Lymphocyte* OR B-Cell Activating Factor* OR Be1 Cell* OR Be2 Cell* OR beta-1 Metal-Binding Globulin OR beta-1B-glycoprotein OR B-Lymphocyte Activating Factor* OR B-Lymphocyte* OR B-Lymphoid* OR C3 Complement* OR C3 Precursor* OR Caeruloplasmin* OR CD 4 OR CD134 Ligand OR CD137 Ligand OR CD153 Antigen* OR CD154 Antigen* OR CD178 Antigen* OR CD253 Antigen* OR CD254 Antigen* OR CD257 Antigen* OR CD30 Ligand OR CD40 Ligand OR CD4-Positive Lymphocyte* OR CD54 Antigen* OR CD70 Antigen* OR CD8 Positive Lymphocyte* OR CD95 Ligand OR Complement 3 OR Complement C3 OR Complement Component 3 OR COX 2 OR D dimer OR D1 dimer OR E Selectin* OR ELAM* OR Endothelial Leukocyte Adhesion Molecule-1 OR Fas Ligand OR fibrin fragment D OR Herpesvirus Entry Mediator-Ligand Protein* OR ICAM* OR Immunoglobulin E OR Immunologic Accessory Cell* OR Intercellular Adhesion Molecule* OR Kazal Pancreatic Trypsin Inhibitor* OR L1 Antigen OR LAK Cell* OR LECAM-2 OR LIGHT Protein* OR Lipocalin-2 OR Lymphokine Activated Killer Cell* OR Neutrophil Gelatinase-Associated Lipocalin OR NGAL Protein* OR Oncogene 24p3 Protein* OR Osteoclast Differentiation Factor* OR OX40 Ligand OR Pancreatic Secretory Trypsin Inhibitor* OR Pro-C3 OR Receptor Activator of Nuclear Factor-kappa* OR Serpin A3 OR Serum A Related Protein OR Serum Amyloid A OR Serum Amyloid P OR Serum Amyloid Protein A OR Serum Sialomucin* OR Siderocalin Protein* OR T cell* OR T Helper OR T Lymphocyte* OR T4 cell* OR T4 Lymphocyte* OR T8 Cell* OR T8 Lymphocyte* OR TALL-1 Protein* OR TALL2 Protein* OR TALL-2 Protein* OR TC1 Cell* OR TC2 Cell* OR Tfh Cell* OR Th1 Cell* OR Th17 Cell* OR Th2 Cell* OR Th3 Cell* OR TL1 Ligand OR TL1A Ligand OR T-Lymphoid* OR TNFS* OR Tr1 Cell* OR TRAIL Protein* OR TRANCE Protein* OR Trypsin Inhibitor Kazal Pancreatic OR Tumor Associated Trypsin Inhibitor* OR tumor necrosis serum* OR tumour necrosis serum* OR Vascular Cell Adhesion Molecule* OR VCAM* OR 8OHdG OR 8-OH-dG OR 8-oxodG OR 8-oxo-dG OR 8-oxodGuo OR 8-oxo-dGuo OR A1PI OR acute phase globulin* OR Acute Phase Glycoprotein* OR acute phase plasma protein* OR acute phase protein* OR Acute Phase Reactant* OR acute phase response protein* OR Amyloid Protein* OR Apo2L OR Apo2-L OR Apo-2-L OR Apo3L OR Apo3-L OR Apo-3-L OR Apoferritin* OR BAFF Ligand OR Basophil* OR Biomarker* OR Blood cell* OR Blood Count* OR Blood Number* OR Bloodcell* OR BLyS Protein OR Breg Cell* OR C Reactive Protein OR Cachectin OR Cachetin OR Calcitonin* OR Calcitrin* OR Calgranulin* OR Calprotectin* OR CD134L OR CD27 Ligand OR CD27L OR CD30L OR CD4 OR CD40L OR CD62E OR CD95L OR Cell Count* OR Cell Number* OR Ceruloplasmin* OR Chemokine* OR Ciba 47175* OR Ciba47175* OR COX2 OR CRP OR CycloOxygenase* OR Cyclo-Oxygenase* OR Cytokine* OR Dendritic Cell* OR Deoxyguanosine* OR Dihydrodeoxyguanosine* OR Dihydroguanosine* OR Ectodysplasin* OR eNO OR Eosinophil* OR Eosinophil* OR Erythrocyte* OR ESR OR Exhaled Nitric Oxide* OR exhaled NO OR Feno OR Ferritin* OR Ferroxidase* OR Fibrinogen* OR Granulocyte* OR Haemopexin* OR Haptoglobin* OR Helper Cell* OR Hematocrit* OR Hemoglobin* OR Hemopexin* OR Hpx OR hsCRP OR HVEML OR HVEM-L OR Hx OR Hydroxydeoxyguanosine* OR IFN OR IgE OR Il OR Il6 OR Inducer Cell* OR Inflammat* OR innate lymphoid cell* OR Intercrine* OR Interdigitating Cell* OR Interferon* OR Interleukin* OR Intraepithelial Lymphocyte* OR Isoferritin* OR Isotransferrin* OR Langerhans Cell* OR LE Cell* OR Leif OR Leucocyte* OR Leukocyte* OR Lymphocyte* OR Lymphotoxin* OR Macrophage* OR MAIT Cell* OR Marker* OR Mast Cell* OR Mastocyte* OR Monocyte* OR Natural Killer Cell* OR neutrophil* OR NK Cell* OR OPGL OR Orosomucoid* OR Osteoprotegerin Ligand OR OX40L OR Oxodeoxyguanosine* OR Oxoguanosine OR Pct OR Plasma cell* OR Plasmacyte* OR Platelet* OR procalcitonin* OR Prolastin OR RANK Ligand OR RANKL OR RbC OR RbCC OR Reticulocyte* OR Seromucoid* OR Serotransferrin* OR Serpin A1 OR Siderophilin* OR Soluble urokinase Plasminogen Activator Receptor* OR SPINK1 OR SuPAR OR THANK Protein OR Thymocyte* OR Thyrocalcitonin* OR tissue necrosis factor* OR TNF OR TNFa* OR Transferrin* OR Treg Cell* OR Tumor Necrosis Factor* OR Tumour Necrosis Factor* OR Veiled Cell* OR WbC OR WbCC OR Zemaira).ti,ab,kw,kf |
| 4 | Animaux | (exp Animals/ NOT exp humans/) OR (rat OR rats OR mouse OR mice OR murine OR Guinea Pig*).ti,ab,kw,kf |
| 5 | Combinaison | 1. AND 2 AND 3 AND (english OR French).lg) NOT 4 |

## Ovid All EBM Reviews

| 1 | Vitamine D | Exp Vitamin D/ OR Vitamin D Deficiency/ OR (25OHD* OR 25OH-D* OR 25-OHD* OR 25-OH-D* OR dht intensol OR Dihydroxyvitamin D* OR Dihydroxyvitamins D* OR dnt intensol OR Hydroxyvitamin D* OR Hydroxyvitamins D* OR MC 1288 OR MC1288 OR vitamin D* OR vitamins D* OR antitanil OR antitetanin* OR atecen OR bocatriol OR calcamin* OR Calcidiol* OR Calcifediol* OR Calciferol* OR calcijex OR calcinosefaktor OR Calciol* OR calcitriol OR Calderol* OR Cholercalciferol* OR decostriol OR Dedrogyl* OR dichistrolum OR dichysterol OR dichystrol OR dihydral OR dihydrotachysterin* OR Dihydrotachysterol* OR dihydroxycholecalciferol* OR DihydroxyvitaminD* OR DihydroxyvitaminsD* OR dikystrol OR dygratyl OR Ercalcidiol* OR Ergocalciferol* OR Hidroferol* OR Hydroxycalciferol* OR Hydroxycholecalciferol* OR Hydroxyergocalciferol* OR HydroxyvitaminD* OR HydroxyvitaminsD* OR hytakerol OR manipal OR osteotriol OR parterol OR renatriol OR silkis OR sitriol OR Soltriol OR tachidon OR tachystin* OR tachystol OR tetilan OR tirocal OR vitaminD* OR vitaminsD*).ti,ab,kw,kf |
| --- | --- | --- |
| 2 | Asthme | Exp Asthma/ OR Asthma*.ti,ab,kw,kf |
| 3 | Biomarqueurs d'inflammation | Biomarkers/ OR Inflammation/ OR Immunoglobulin E/ OR exp C-Reactive Protein/ OR exp Calcitonin/ OR Exp Procalcitonin/ OR exp Cell Count/ OR exp Blood Cells/ OR exp Fractional Exhaled Nitric Oxide Testing/ OR Exp Hematocrit/ OR Exp Cytokines/ OR Hemoglobins/ OR Exp Blood Sedimentation/ OR Exp Leukocyte L1 Antigen Complex/ OR Exp Ferritins/ OR Exp Fibrinogen/ OR Exp Serum Amyloid A Protein/ OR Exp 8-Hydroxy-2'-Deoxyguanosine/ OR Exp Cyclooxygenase 2/ OR Exp Receptors, Urokinase Plasminogen Activator/ OR Exp Mast Cells/ OR Macrophages/ OR Exp Acute-Phase Proteins/ OR Exp Interleukins/ OR Exp Tumor Necrosis Factors/ OR Exp E-Selectin/ OR Exp Intercellular Adhesion Molecule-1/ OR Exp Vascular Cell Adhesion Molecule-1/ OR Exp Interferons/ OR Exp Dendritic Cells/ OR (Exp Immunity, Innate/ AND Exp Lymphocytes/) OR Exp Killer Cells, Natural/ OR Exp T-Lymphocytes/ OR Exp B-Lymphocytes/ OR Exp Antigen-Presenting Cells/ OR (27E10 Antigen OR 4-1BB Ligand OR 4-1BBL OR 45S RNP OR A Proliferation Inducing Ligand Protein* OR Acid alpha 1-Glycoprotein* OR Acidic Pancreatic Trypsin Inhibitor* OR alpha 1 Antiprotease OR alpha 1 Antitrypsin OR alpha 1-Acid Glycoprotein* OR alpha 1-Antichymotrypsin* OR alpha 1-Antiproteinase OR alpha 1-Glycoprotein Acid* OR alpha 1-Macroglobulin* OR alpha 1-Protease Inhibitor OR alpha 1-Proteinase Inhibitor OR alpha 2-Globulin* OR alpha 2-Macroglobulin* OR alpha Macroglobulin* OR alpha-Macrofetoprotein* OR Amyloid A Precursor OR Amyloid A Protein OR Amyloid A OR Amyloid Fibril Protein AA OR Amyloid P Component* OR Amyloid Protein AA OR Amyloid Protein AA OR Amyloid Protein SAA OR Amyloid Serum Protein SAA OR Antigen L1 OR Antigen-Presenting Cell* OR Apo 2 Ligand OR Apo 3 Ligand OR Apo2 Ligand OR Apo3 Ligand OR APRIL Protein* OR B cell* OR B Lymphocyte Stimulator* OR B1 Cell* OR B-1 Cell* OR B1 Lymphocyte* OR B-1 Lymphocyte* OR B2 Cell* OR B-2 Cell* OR B2 Lymphocyte* OR B-2 Lymphocyte* OR B-Cell Activating Factor* OR Be1 Cell* OR Be2 Cell* OR beta-1 Metal-Binding Globulin OR beta-1B-glycoprotein OR B-Lymphocyte Activating Factor* OR B-Lymphocyte* OR B-Lymphoid* OR C3 Complement* OR C3 Precursor* OR Caeruloplasmin* OR CD 4 OR CD134 Ligand OR CD137 Ligand OR CD153 Antigen* OR CD154 Antigen* OR CD178 Antigen* OR CD253 Antigen* OR CD254 Antigen* OR CD257 Antigen* OR CD30 Ligand OR CD40 Ligand OR CD4-Positive Lymphocyte* OR CD54 Antigen* OR CD70 Antigen* OR CD8 Positive Lymphocyte* OR CD95 Ligand OR Complement 3 OR Complement C3 OR Complement Component 3 OR COX 2 OR D dimer OR D1 dimer OR E Selectin* OR ELAM* OR Endothelial Leukocyte Adhesion Molecule-1 OR Fas Ligand OR fibrin fragment D OR Herpesvirus Entry Mediator-Ligand Protein* OR ICAM* OR Immunoglobulin E OR Immunologic Accessory Cell* OR Intercellular Adhesion Molecule* OR Kazal Pancreatic Trypsin Inhibitor* OR L1 Antigen OR LAK Cell* OR LECAM-2 OR LIGHT Protein* OR Lipocalin-2 OR Lymphokine Activated Killer Cell* OR Neutrophil Gelatinase-Associated Lipocalin OR NGAL Protein* OR Oncogene 24p3 Protein* OR Osteoclast Differentiation Factor* OR OX40 Ligand OR Pancreatic Secretory Trypsin Inhibitor* OR Pro-C3 OR Receptor Activator of Nuclear Factor-kappa* OR Serpin A3 OR Serum A Related Protein OR Serum Amyloid A OR Serum Amyloid P OR Serum Amyloid Protein A OR Serum Sialomucin* OR Siderocalin Protein* OR T cell* OR T Helper OR T Lymphocyte* OR T4 cell* OR T4 Lymphocyte* OR T8 Cell* OR T8 Lymphocyte* OR TALL-1 Protein* OR TALL2 Protein* OR TALL-2 Protein* OR TC1 Cell* OR TC2 Cell* OR Tfh Cell* OR Th1 Cell* OR Th17 Cell* OR Th2 Cell* OR Th3 Cell* OR TL1 Ligand OR TL1A Ligand OR T-Lymphoid* OR TNFS* OR Tr1 Cell* OR TRAIL Protein* OR TRANCE Protein* OR Trypsin Inhibitor Kazal Pancreatic OR Tumor Associated Trypsin Inhibitor* OR tumor necrosis serum* OR tumour necrosis serum* OR Vascular Cell Adhesion Molecule* OR VCAM* OR 8OHdG OR 8-OH-dG OR 8-oxodG OR 8-oxo-dG OR 8-oxodGuo OR 8-oxo-dGuo OR A1PI OR acute phase globulin* OR Acute Phase Glycoprotein* OR acute phase plasma protein* OR acute phase protein* OR Acute Phase Reactant* OR acute phase response protein* OR Amyloid Protein* OR Apo2L OR Apo2-L OR Apo-2-L OR Apo3L OR Apo3-L OR Apo-3-L OR Apoferritin* OR BAFF Ligand OR Basophil* OR Biomarker* OR Blood cell* OR Blood Count* OR Blood Number* OR Bloodcell* OR BLyS Protein OR Breg Cell* OR C Reactive Protein OR Cachectin OR Cachetin OR Calcitonin* OR Calcitrin* OR Calgranulin* OR Calprotectin* OR CD134L OR CD27 Ligand OR CD27L OR CD30L OR CD4 OR CD40L OR CD62E OR CD95L OR Cell Count* OR Cell Number* OR Ceruloplasmin* OR Chemokine* OR Ciba 47175* OR Ciba47175* OR COX2 OR CRP OR CycloOxygenase* OR Cyclo-Oxygenase* OR Cytokine* OR Dendritic Cell* OR Deoxyguanosine* OR Dihydrodeoxyguanosine* OR Dihydroguanosine* OR Ectodysplasin* OR eNO OR Eosinophil* OR Eosinophil* OR Erythrocyte* OR ESR OR Exhaled Nitric Oxide* OR exhaled NO OR Feno OR Ferritin* OR Ferroxidase* OR Fibrinogen* OR Granulocyte* OR Haemopexin* OR Haptoglobin* OR Helper Cell* OR Hematocrit* OR Hemoglobin* OR Hemopexin* OR Hpx OR hsCRP OR HVEML OR HVEM-L OR Hx OR Hydroxydeoxyguanosine* OR IFN OR IgE OR Il OR Il6 OR Inducer Cell* OR Inflammat* OR innate lymphoid cell* OR Intercrine* OR Interdigitating Cell* OR Interferon* OR Interleukin* OR Intraepithelial Lymphocyte* OR Isoferritin* OR Isotransferrin* OR Langerhans Cell* OR LE Cell* OR Leif OR Leucocyte* OR Leukocyte* OR Lymphocyte* OR Lymphotoxin* OR Macrophage* OR MAIT Cell* OR Marker* OR Mast Cell* OR Mastocyte* OR Monocyte* OR Natural Killer Cell* OR neutrophil* OR NK Cell* OR OPGL OR Orosomucoid* OR Osteoprotegerin Ligand OR OX40L OR Oxodeoxyguanosine* OR Oxoguanosine OR Pct OR Plasma cell* OR Plasmacyte* OR Platelet* OR procalcitonin* OR Prolastin OR RANK Ligand OR RANKL OR RbC OR RbCC OR Reticulocyte* OR Seromucoid* OR Serotransferrin* OR Serpin A1 OR Siderophilin* OR Soluble urokinase Plasminogen Activator Receptor* OR SPINK1 OR SuPAR OR THANK Protein OR Thymocyte* OR Thyrocalcitonin* OR tissue necrosis factor* OR TNF OR TNFa* OR Transferrin* OR Treg Cell* OR Tumor Necrosis Factor* OR Tumour Necrosis Factor* OR Veiled Cell* OR WbC OR WbCC OR Zemaira).ti,ab,kw,kf |
| 4 | Animaux | (exp Animals/ NOT exp humans/) OR (rat OR rats OR mouse OR mice OR murine OR Guinea Pig*).ti,ab,kw,kf |
| 5 | Combinaison | 1. AND 2 AND 3 AND (english OR French).lg) NOT 4 |

## Ovid Embase

| 1 | Vitamine D | Exp Vitamin D/ OR Vitamin D Deficiency/ OR (25OHD* OR 25OH-D* OR 25-OHD* OR 25-OH-D* OR dht intensol OR Dihydroxyvitamin D* OR Dihydroxyvitamins D* OR dnt intensol OR Hydroxyvitamin D* OR Hydroxyvitamins D* OR MC 1288 OR MC1288 OR vitamin D* OR vitamins D* OR antitanil OR antitetanin* OR atecen OR bocatriol OR calcamin* OR Calcidiol* OR Calcifediol* OR Calciferol* OR calcijex OR calcinosefaktor OR Calciol* OR calcitriol OR Calderol* OR Cholercalciferol* OR decostriol OR Dedrogyl* OR dichistrolum OR dichysterol OR dichystrol OR dihydral OR dihydrotachysterin* OR Dihydrotachysterol* OR dihydroxycholecalciferol* OR DihydroxyvitaminD* OR DihydroxyvitaminsD* OR dikystrol OR dygratyl OR Ercalcidiol* OR Ergocalciferol* OR Hidroferol* OR Hydroxycalciferol* OR Hydroxycholecalciferol* OR Hydroxyergocalciferol* OR HydroxyvitaminD* OR HydroxyvitaminsD* OR hytakerol OR manipal OR osteotriol OR parterol OR renatriol OR silkis OR sitriol OR Soltriol OR tachidon OR tachystin* OR tachystol OR tetilan OR tirocal OR vitaminD* OR vitaminsD*).ti,ab,kw |
| --- | --- | --- |
| 2 | Asthme | Exp Asthma/ OR Asthma*.ti,ab,kw |
| 3 | Biomarqueurs d'inflammation | biological marker/ OR Inflammation/ OR Immunoglobulin E/ OR C Reactive Protein/ OR Calcitonin/ OR Procalcitonin/ OR exp Cell Count/ OR exp Blood Cell/ OR fractional exhaled nitric oxide test/ OR Hematocrit/ OR Exp Cytokine/ OR Hemoglobin/ OR erythrocyte sedimentation rate/ OR calgranulin/ OR Ferritin/ OR Fibrinogen/ OR Serum Amyloid A/ OR 8 hydroxydeoxyguanosine/ OR Exp Cyclooxygenase 2/ OR Urokinase Receptor/ OR Exp Mast Cell/ OR Macrophage/ OR Acute Phase Protein/ OR endothelial leukocyte adhesion molecule 1/ OR intercellular adhesion molecule 1/ OR vascular cell adhesion molecule 1/ OR Exp Dendritic Cell/ OR exp innate lymphoid cell/ OR Exp T Lymphocyte/ OR Exp B Lymphocyte/ OR Antigen Presenting Cell/ OR (27E10 Antigen OR 4-1BB Ligand OR 4-1BBL OR 45S RNP OR A Proliferation Inducing Ligand Protein* OR Acid alpha 1-Glycoprotein* OR Acidic Pancreatic Trypsin Inhibitor* OR alpha 1 Antiprotease OR alpha 1 Antitrypsin OR alpha 1-Acid Glycoprotein* OR alpha 1-Antichymotrypsin* OR alpha 1-Antiproteinase OR alpha 1-Glycoprotein Acid* OR alpha 1-Macroglobulin* OR alpha 1-Protease Inhibitor OR alpha 1-Proteinase Inhibitor OR alpha 2-Globulin* OR alpha 2-Macroglobulin* OR alpha Macroglobulin* OR alpha-Macrofetoprotein* OR Amyloid A Precursor OR Amyloid A Protein OR Amyloid A OR Amyloid Fibril Protein AA OR Amyloid P Component* OR Amyloid Protein AA OR Amyloid Protein AA OR Amyloid Protein SAA OR Amyloid Serum Protein SAA OR Antigen L1 OR Antigen-Presenting Cell* OR Apo 2 Ligand OR Apo 3 Ligand OR Apo2 Ligand OR Apo3 Ligand OR APRIL Protein* OR B cell* OR B Lymphocyte Stimulator* OR B1 Cell* OR B-1 Cell* OR B1 Lymphocyte* OR B-1 Lymphocyte* OR B2 Cell* OR B-2 Cell* OR B2 Lymphocyte* OR B-2 Lymphocyte* OR B-Cell Activating Factor* OR Be1 Cell* OR Be2 Cell* OR beta-1 Metal-Binding Globulin OR beta-1B-glycoprotein OR B-Lymphocyte Activating Factor* OR B-Lymphocyte* OR B-Lymphoid* OR C3 Complement* OR C3 Precursor* OR Caeruloplasmin* OR CD 4 OR CD134 Ligand OR CD137 Ligand OR CD153 Antigen* OR CD154 Antigen* OR CD178 Antigen* OR CD253 Antigen* OR CD254 Antigen* OR CD257 Antigen* OR CD30 Ligand OR CD40 Ligand OR CD4-Positive Lymphocyte* OR CD54 Antigen* OR CD70 Antigen* OR CD8 Positive Lymphocyte* OR CD95 Ligand OR Complement 3 OR Complement C3 OR Complement Component 3 OR COX 2 OR D dimer OR D1 dimer OR E Selectin* OR ELAM* OR Endothelial Leukocyte Adhesion Molecule-1 OR Fas Ligand OR fibrin fragment D OR Herpesvirus Entry Mediator-Ligand Protein* OR ICAM* OR Immunoglobulin E OR Immunologic Accessory Cell* OR Intercellular Adhesion Molecule* OR Kazal Pancreatic Trypsin Inhibitor* OR L1 Antigen OR LAK Cell* OR LECAM-2 OR LIGHT Protein* OR Lipocalin-2 OR Lymphokine Activated Killer Cell* OR Neutrophil Gelatinase-Associated Lipocalin OR NGAL Protein* OR Oncogene 24p3 Protein* OR Osteoclast Differentiation Factor* OR OX40 Ligand OR Pancreatic Secretory Trypsin Inhibitor* OR Pro-C3 OR Receptor Activator of Nuclear Factor-kappa* OR Serpin A3 OR Serum A Related Protein OR Serum Amyloid A OR Serum Amyloid P OR Serum Amyloid Protein A OR Serum Sialomucin* OR Siderocalin Protein* OR T cell* OR T Helper OR T Lymphocyte* OR T4 cell* OR T4 Lymphocyte* OR T8 Cell* OR T8 Lymphocyte* OR TALL-1 Protein* OR TALL2 Protein* OR TALL-2 Protein* OR TC1 Cell* OR TC2 Cell* OR Tfh Cell* OR Th1 Cell* OR Th17 Cell* OR Th2 Cell* OR Th3 Cell* OR TL1 Ligand OR TL1A Ligand OR T-Lymphoid* OR TNFS* OR Tr1 Cell* OR TRAIL Protein* OR TRANCE Protein* OR Trypsin Inhibitor Kazal Pancreatic OR Tumor Associated Trypsin Inhibitor* OR tumor necrosis serum* OR tumour necrosis serum* OR Vascular Cell Adhesion Molecule* OR VCAM* OR 8OHdG OR 8-OH-dG OR 8-oxodG OR 8-oxo-dG OR 8-oxodGuo OR 8-oxo-dGuo OR A1PI OR acute phase globulin* OR Acute Phase Glycoprotein* OR acute phase plasma protein* OR acute phase protein* OR Acute Phase Reactant* OR acute phase response protein* OR Amyloid Protein* OR Apo2L OR Apo2-L OR Apo-2-L OR Apo3L OR Apo3-L OR Apo-3-L OR Apoferritin* OR BAFF Ligand OR Basophil* OR Biomarker* OR Blood cell* OR Blood Count* OR Blood Number* OR Bloodcell* OR BLyS Protein OR Breg Cell* OR C Reactive Protein OR Cachectin OR Cachetin OR Calcitonin* OR Calcitrin* OR Calgranulin* OR Calprotectin* OR CD134L OR CD27 Ligand OR CD27L OR CD30L OR CD4 OR CD40L OR CD62E OR CD95L OR Cell Count* OR Cell Number* OR Ceruloplasmin* OR Chemokine* OR Ciba 47175* OR Ciba47175* OR COX2 OR CRP OR CycloOxygenase* OR Cyclo-Oxygenase* OR Cytokine* OR Dendritic Cell* OR Deoxyguanosine* OR Dihydrodeoxyguanosine* OR Dihydroguanosine* OR Ectodysplasin* OR eNO OR Eosinophil* OR Eosinophil* OR Erythrocyte* OR ESR OR Exhaled Nitric Oxide* OR exhaled NO OR Feno OR Ferritin* OR Ferroxidase* OR Fibrinogen* OR Granulocyte* OR Haemopexin* OR Haptoglobin* OR Helper Cell* OR Hematocrit* OR Hemoglobin* OR Hemopexin* OR Hpx OR hsCRP OR HVEML OR HVEM-L OR Hx OR Hydroxydeoxyguanosine* OR IFN OR IgE OR Il OR Il6 OR Inducer Cell* OR Inflammat* OR innate lymphoid cell* OR Intercrine* OR Interdigitating Cell* OR Interferon* OR Interleukin* OR Intraepithelial Lymphocyte* OR Isoferritin* OR Isotransferrin* OR Langerhans Cell* OR LE Cell* OR Leif OR Leucocyte* OR Leukocyte* OR Lymphocyte* OR Lymphotoxin* OR Macrophage* OR MAIT Cell* OR Marker* OR Mast Cell* OR Mastocyte* OR Monocyte* OR Natural Killer Cell* OR neutrophil* OR NK Cell* OR OPGL OR Orosomucoid* OR Osteoprotegerin Ligand OR OX40L OR Oxodeoxyguanosine* OR Oxoguanosine OR Pct OR Plasma cell* OR Plasmacyte* OR Platelet* OR procalcitonin* OR Prolastin OR RANK Ligand OR RANKL OR RbC OR RbCC OR Reticulocyte* OR Seromucoid* OR Serotransferrin* OR Serpin A1 OR Siderophilin* OR Soluble urokinase Plasminogen Activator Receptor* OR SPINK1 OR SuPAR OR THANK Protein OR Thymocyte* OR Thyrocalcitonin* OR tissue necrosis factor* OR TNF OR TNFa* OR Transferrin* OR Treg Cell* OR Tumor Necrosis Factor* OR Tumour Necrosis Factor* OR Veiled Cell* OR WbC OR WbCC OR Zemaira).ti,ab,kw |
| 4 | Animaux | (exp Animal/ NOT exp human/) OR (rat OR rats OR mouse OR mice OR murine OR Guinea Pig*).ti,ab,kw |
| 5 | Combinaison | 1. AND 2 AND 3 AND (english OR French).lg) NOT 4 |

## CINAHL EBSCO

| S1 | Vitamine D | MH(Vitamin D+ OR Vitamin D Deficiency) OR TI(25OHD* OR 25OH-D* OR 25-OHD* OR 25-OH-D* OR dht intensol OR Dihydroxyvitamin D* OR Dihydroxyvitamins D* OR dnt intensol OR Hydroxyvitamin D* OR Hydroxyvitamins D* OR MC 1288 OR MC1288 OR vitamin D* OR vitamins D* OR antitanil OR antitetanin* OR atecen OR bocatriol OR calcamin* OR Calcidiol* OR Calcifediol* OR Calciferol* OR calcijex OR calcinosefaktor OR Calciol* OR calcitriol OR Calderol* OR Cholercalciferol* OR decostriol OR Dedrogyl* OR dichistrolum OR dichysterol OR dichystrol OR dihydral OR dihydrotachysterin* OR Dihydrotachysterol* OR dihydroxycholecalciferol* OR DihydroxyvitaminD* OR DihydroxyvitaminsD* OR dikystrol OR dygratyl OR Ercalcidiol* OR Ergocalciferol* OR Hidroferol* OR Hydroxycalciferol* OR Hydroxycholecalciferol* OR Hydroxyergocalciferol* OR HydroxyvitaminD* OR HydroxyvitaminsD* OR hytakerol OR manipal OR osteotriol OR parterol OR renatriol OR silkis OR sitriol OR Soltriol OR tachidon OR tachystin* OR tachystol OR tetilan OR tirocal OR vitaminD* OR vitaminsD*) OR AB(25OHD* OR 25OH-D* OR 25-OHD* OR 25-OH-D* OR dht intensol OR Dihydroxyvitamin D* OR Dihydroxyvitamins D* OR dnt intensol OR Hydroxyvitamin D* OR Hydroxyvitamins D* OR MC 1288 OR MC1288 OR vitamin D* OR vitamins D* OR antitanil OR antitetanin* OR atecen OR bocatriol OR calcamin* OR Calcidiol* OR Calcifediol* OR Calciferol* OR calcijex OR calcinosefaktor OR Calciol* OR calcitriol OR Calderol* OR Cholercalciferol* OR decostriol OR Dedrogyl* OR dichistrolum OR dichysterol OR dichystrol OR dihydral OR dihydrotachysterin* OR Dihydrotachysterol* OR dihydroxycholecalciferol* OR DihydroxyvitaminD* OR DihydroxyvitaminsD* OR dikystrol OR dygratyl OR Ercalcidiol* OR Ergocalciferol* OR Hidroferol* OR Hydroxycalciferol* OR Hydroxycholecalciferol* OR Hydroxyergocalciferol* OR HydroxyvitaminD* OR HydroxyvitaminsD* OR hytakerol OR manipal OR osteotriol OR parterol OR renatriol OR silkis OR sitriol OR Soltriol OR tachidon OR tachystin* OR tachystol OR tetilan OR tirocal OR vitaminD* OR vitaminsD*) |
| --- | --- | --- |
| S2 | Asthme | MH(Asthma+) OR TI(Asthma*) OR AB(Asthma*) |
| S3 | Biomarqueurs d'inflammation | MH(Biological Markers OR Inflammation OR Immunoglobulins OR C-Reactive Protein OR  Calcitonin OR Cell Count+ OR Blood Cells+ OR Fractional Exhaled Nitric Oxide Testing OR Hematocrit OR Cytokines+ OR Hemoglobins OR Blood Sedimentation OR Ferritin OR Fibrinogen+ OR Mast Cells OR Macrophages OR Acute-Phase Proteins+ OR Interleukins+ OR Tumor Necrosis Factor OR Interferons OR Dendritic Cells OR "Killer Cells, Natural" OR T Lymphocytes+ OR B Lymphocytes+ OR Antigen-Presenting Cells) OR TI("27E10 Antigen" OR "4-1BB Ligand" OR "4-1BBL" OR "45S RNP" OR "A Proliferation Inducing Ligand Protein*" OR "Acid alpha 1-Glycoprotein*" OR "Acidic Pancreatic Trypsin Inhibitor*" OR "alpha 1 Antiprotease" OR "alpha 1 Antitrypsin" OR "alpha 1-Acid Glycoprotein*" OR "alpha 1-Antichymotrypsin*" OR "alpha 1-Antiproteinase" OR "alpha 1-Glycoprotein Acid*" OR "alpha 1-Macroglobulin*" OR "alpha 1-Protease Inhibitor" OR "alpha 1-Proteinase Inhibitor" OR "alpha 2-Globulin*" OR "alpha 2-Macroglobulin*" OR "alpha Macroglobulin*" OR "alpha-Macrofetoprotein*" OR "Amyloid A Precursor" OR "Amyloid A Protein" OR "Amyloid A" OR "Amyloid Fibril Protein AA" OR "Amyloid P Component*" OR "Amyloid Protein AA" OR "Amyloid Protein AA" OR "Amyloid Protein SAA" OR "Amyloid Serum Protein SAA" OR "Antigen L1" OR "Antigen-Presenting Cell*" OR "Apo 2 Ligand" OR "Apo 3 Ligand" OR "Apo2 Ligand" OR "Apo3 Ligand" OR "APRIL Protein*" OR "B cell*" OR "B Lymphocyte Stimulator*" OR "B1 Cell*" OR "B-1 Cell*" OR "B1 Lymphocyte*" OR "B-1 Lymphocyte*" OR "B2 Cell*" OR "B-2 Cell*" OR "B2 Lymphocyte*" OR "B-2 Lymphocyte*" OR "B-Cell Activating Factor*" OR "Be1 Cell*" OR "Be2 Cell*" OR "beta-1 Metal-Binding Globulin" OR "beta-1B-glycoprotein" OR "B-Lymphocyte Activating Factor*" OR "B-Lymphocyte*" OR "B-Lymphoid*" OR "C3 Complement*" OR "C3 Precursor*" OR "Caeruloplasmin*" OR "CD 4" OR "CD134 Ligand" OR "CD137 Ligand" OR "CD153 Antigen*" OR "CD154 Antigen*" OR "CD178 Antigen*" OR "CD253 Antigen*" OR "CD254 Antigen*" OR "CD257 Antigen*" OR "CD30 Ligand" OR "CD40 Ligand" OR "CD4-Positive Lymphocyte*" OR "CD54 Antigen*" OR "CD70 Antigen*" OR "CD8 Positive Lymphocyte*" OR "CD95 Ligand" OR "Complement 3" OR "Complement C3" OR "Complement Component 3" OR "COX 2" OR "D dimer" OR "D1 dimer" OR "E Selectin*" OR "ELAM*" OR "Endothelial Leukocyte Adhesion Molecule-1" OR "Fas Ligand" OR "fibrin fragment D" OR "Herpesvirus Entry Mediator-Ligand Protein*" OR "ICAM*" OR "Immunoglobulin E" OR "Immunologic Accessory Cell*" OR "Intercellular Adhesion Molecule*" OR "Kazal Pancreatic Trypsin Inhibitor*" OR "L1 Antigen" OR "LAK Cell*" OR "LECAM-2" OR "LIGHT Protein*" OR "Lipocalin-2" OR "Lymphokine Activated Killer Cell*" OR "Neutrophil Gelatinase-Associated Lipocalin" OR "NGAL Protein*" OR "Oncogene 24p3 Protein*" OR "Osteoclast Differentiation Factor*" OR "OX40 Ligand" OR "Pancreatic Secretory Trypsin Inhibitor*" OR "Pro-C3" OR "Receptor Activator of Nuclear Factor-kappa*" OR "Serpin A3" OR "Serum A Related Protein" OR "Serum Amyloid A" OR "Serum Amyloid P" OR "Serum Amyloid Protein A" OR "Serum Sialomucin*" OR "Siderocalin Protein*" OR "T cell*" OR "T Helper" OR "T Lymphocyte*" OR "T4 cell*" OR "T4 Lymphocyte*" OR "T8 Cell*" OR "T8 Lymphocyte*" OR "TALL-1 Protein*" OR "TALL2 Protein*" OR "TALL-2 Protein*" OR "TC1 Cell*" OR "TC2 Cell*" OR "Tfh Cell*" OR "Th1 Cell*" OR "Th17 Cell*" OR "Th2 Cell*" OR "Th3 Cell*" OR "TL1 Ligand" OR "TL1A Ligand" OR "T-Lymphoid*" OR "TNFS*" OR "Tr1 Cell*" OR "TRAIL Protein*" OR "TRANCE Protein*" OR "Trypsin Inhibitor Kazal Pancreatic" OR "Tumor Associated Trypsin Inhibitor*" OR "tumor necrosis serum*" OR "tumour necrosis serum*" OR "Vascular Cell Adhesion Molecule*" OR "VCAM*" OR 8OHdG OR 8-OH-dG OR 8-oxodG OR 8-oxo-dG OR 8-oxodGuo OR 8-oxo-dGuo OR A1PI OR acute phase globulin* OR Acute Phase Glycoprotein* OR acute phase plasma protein* OR acute phase protein* OR Acute Phase Reactant* OR acute phase response protein* OR Amyloid Protein* OR Apo2L OR Apo2-L OR "Apo-2-L" OR Apo3L OR Apo3-L OR "Apo-3-L" OR Apoferritin* OR BAFF Ligand OR Basophil* OR Biomarker* OR Blood cell* OR Blood Count* OR Blood Number* OR Bloodcell* OR BLyS Protein OR Breg Cell* OR C Reactive Protein OR Cachectin OR Cachetin OR Calcitonin* OR Calcitrin* OR Calgranulin* OR Calprotectin* OR CD134L OR CD27 Ligand OR CD27L OR CD30L OR CD4 OR CD40L OR CD62E OR CD95L OR Cell Count* OR Cell Number* OR Ceruloplasmin* OR Chemokine* OR Ciba 47175* OR Ciba47175* OR COX2 OR CRP OR CycloOxygenase* OR Cyclo-Oxygenase* OR Cytokine* OR Dendritic Cell* OR Deoxyguanosine* OR Dihydrodeoxyguanosine* OR Dihydroguanosine* OR Ectodysplasin* OR eNO OR Eosinophil* OR Eosinophil* OR Erythrocyte* OR ESR OR Exhaled Nitric Oxide* OR exhaled NO OR Feno OR Ferritin* OR Ferroxidase* OR Fibrinogen* OR Granulocyte* OR Haemopexin* OR Haptoglobin* OR Helper Cell* OR Hematocrit* OR Hemoglobin* OR Hemopexin* OR Hpx OR hsCRP OR HVEML OR HVEM-L OR Hx OR Hydroxydeoxyguanosine* OR IFN OR IgE OR Il OR Il6 OR Inducer Cell* OR Inflammat* OR innate lymphoid cell* OR Intercrine* OR Interdigitating Cell* OR Interferon* OR Interleukin* OR Intraepithelial Lymphocyte* OR Isoferritin* OR Isotransferrin* OR Langerhans Cell* OR LE Cell* OR Leif OR Leucocyte* OR Leukocyte* OR Lymphocyte* OR Lymphotoxin* OR Macrophage* OR MAIT Cell* OR Marker* OR Mast Cell* OR Mastocyte* OR Monocyte* OR Natural Killer Cell* OR neutrophil* OR NK Cell* OR OPGL OR Orosomucoid* OR Osteoprotegerin Ligand OR OX40L OR Oxodeoxyguanosine* OR Oxoguanosine OR Pct OR Plasma cell* OR Plasmacyte* OR Platelet* OR procalcitonin* OR Prolastin OR RANK Ligand OR RANKL OR RbC OR RbCC OR Reticulocyte* OR Seromucoid* OR Serotransferrin* OR Serpin A1 OR Siderophilin* OR Soluble urokinase Plasminogen Activator Receptor* OR SPINK1 OR SuPAR OR THANK Protein OR Thymocyte* OR Thyrocalcitonin* OR tissue necrosis factor* OR TNF OR TNFa* OR Transferrin* OR Treg Cell* OR Tumor Necrosis Factor* OR Tumour Necrosis Factor* OR Veiled Cell* OR WbC OR WbCC OR Zemaira) OR AB("27E10 Antigen" OR "4-1BB Ligand" OR "4-1BBL" OR "45S RNP" OR "A Proliferation Inducing Ligand Protein*" OR "Acid alpha 1-Glycoprotein*" OR "Acidic Pancreatic Trypsin Inhibitor*" OR "alpha 1 Antiprotease" OR "alpha 1 Antitrypsin" OR "alpha 1-Acid Glycoprotein*" OR "alpha 1-Antichymotrypsin*" OR "alpha 1-Antiproteinase" OR "alpha 1-Glycoprotein Acid*" OR "alpha 1-Macroglobulin*" OR "alpha 1-Protease Inhibitor" OR "alpha 1-Proteinase Inhibitor" OR "alpha 2-Globulin*" OR "alpha 2-Macroglobulin*" OR "alpha Macroglobulin*" OR "alpha-Macrofetoprotein*" OR "Amyloid A Precursor" OR "Amyloid A Protein" OR "Amyloid A" OR "Amyloid Fibril Protein AA" OR "Amyloid P Component*" OR "Amyloid Protein AA" OR "Amyloid Protein AA" OR "Amyloid Protein SAA" OR "Amyloid Serum Protein SAA" OR "Antigen L1" OR "Antigen-Presenting Cell*" OR "Apo 2 Ligand" OR "Apo 3 Ligand" OR "Apo2 Ligand" OR "Apo3 Ligand" OR "APRIL Protein*" OR "B cell*" OR "B Lymphocyte Stimulator*" OR "B1 Cell*" OR "B-1 Cell*" OR "B1 Lymphocyte*" OR "B-1 Lymphocyte*" OR "B2 Cell*" OR "B-2 Cell*" OR "B2 Lymphocyte*" OR "B-2 Lymphocyte*" OR "B-Cell Activating Factor*" OR "Be1 Cell*" OR "Be2 Cell*" OR "beta-1 Metal-Binding Globulin" OR "beta-1B-glycoprotein" OR "B-Lymphocyte Activating Factor*" OR "B-Lymphocyte*" OR "B-Lymphoid*" OR "C3 Complement*" OR "C3 Precursor*" OR "Caeruloplasmin*" OR "CD 4" OR "CD134 Ligand" OR "CD137 Ligand" OR "CD153 Antigen*" OR "CD154 Antigen*" OR "CD178 Antigen*" OR "CD253 Antigen*" OR "CD254 Antigen*" OR "CD257 Antigen*" OR "CD30 Ligand" OR "CD40 Ligand" OR "CD4-Positive Lymphocyte*" OR "CD54 Antigen*" OR "CD70 Antigen*" OR "CD8 Positive Lymphocyte*" OR "CD95 Ligand" OR "Complement 3" OR "Complement C3" OR "Complement Component 3" OR "COX 2" OR "D dimer" OR "D1 dimer" OR "E Selectin*" OR "ELAM*" OR "Endothelial Leukocyte Adhesion Molecule-1" OR "Fas Ligand" OR "fibrin fragment D" OR "Herpesvirus Entry Mediator-Ligand Protein*" OR "ICAM*" OR "Immunoglobulin E" OR "Immunologic Accessory Cell*" OR "Intercellular Adhesion Molecule*" OR "Kazal Pancreatic Trypsin Inhibitor*" OR "L1 Antigen" OR "LAK Cell*" OR "LECAM-2" OR "LIGHT Protein*" OR "Lipocalin-2" OR "Lymphokine Activated Killer Cell*" OR "Neutrophil Gelatinase-Associated Lipocalin" OR "NGAL Protein*" OR "Oncogene 24p3 Protein*" OR "Osteoclast Differentiation Factor*" OR "OX40 Ligand" OR "Pancreatic Secretory Trypsin Inhibitor*" OR "Pro-C3" OR "Receptor Activator of Nuclear Factor-kappa*" OR "Serpin A3" OR "Serum A Related Protein" OR "Serum Amyloid A" OR "Serum Amyloid P" OR "Serum Amyloid Protein A" OR "Serum Sialomucin*" OR "Siderocalin Protein*" OR "T cell*" OR "T Helper" OR "T Lymphocyte*" OR "T4 cell*" OR "T4 Lymphocyte*" OR "T8 Cell*" OR "T8 Lymphocyte*" OR "TALL-1 Protein*" OR "TALL2 Protein*" OR "TALL-2 Protein*" OR "TC1 Cell*" OR "TC2 Cell*" OR "Tfh Cell*" OR "Th1 Cell*" OR "Th17 Cell*" OR "Th2 Cell*" OR "Th3 Cell*" OR "TL1 Ligand" OR "TL1A Ligand" OR "T-Lymphoid*" OR "TNFS*" OR "Tr1 Cell*" OR "TRAIL Protein*" OR "TRANCE Protein*" OR "Trypsin Inhibitor Kazal Pancreatic" OR "Tumor Associated Trypsin Inhibitor*" OR "tumor necrosis serum*" OR "tumour necrosis serum*" OR "Vascular Cell Adhesion Molecule*" OR "VCAM*" OR 8OHdG OR 8-OH-dG OR 8-oxodG OR 8-oxo-dG OR 8-oxodGuo OR 8-oxo-dGuo OR A1PI OR acute phase globulin* OR Acute Phase Glycoprotein* OR acute phase plasma protein* OR acute phase protein* OR Acute Phase Reactant* OR acute phase response protein* OR Amyloid Protein* OR Apo2L OR Apo2-L OR "Apo-2-L" OR Apo3L OR Apo3-L OR "Apo-3-L" OR Apoferritin* OR BAFF Ligand OR Basophil* OR Biomarker* OR Blood cell* OR Blood Count* OR Blood Number* OR Bloodcell* OR BLyS Protein OR Breg Cell* OR C Reactive Protein OR Cachectin OR Cachetin OR Calcitonin* OR Calcitrin* OR Calgranulin* OR Calprotectin* OR CD134L OR CD27 Ligand OR CD27L OR CD30L OR CD4 OR CD40L OR CD62E OR CD95L OR Cell Count* OR Cell Number* OR Ceruloplasmin* OR Chemokine* OR Ciba 47175* OR Ciba47175* OR COX2 OR CRP OR CycloOxygenase* OR Cyclo-Oxygenase* OR Cytokine* OR Dendritic Cell* OR Deoxyguanosine* OR Dihydrodeoxyguanosine* OR Dihydroguanosine* OR Ectodysplasin* OR eNO OR Eosinophil* OR Eosinophil* OR Erythrocyte* OR ESR OR Exhaled Nitric Oxide* OR exhaled NO OR Feno OR Ferritin* OR Ferroxidase* OR Fibrinogen* OR Granulocyte* OR Haemopexin* OR Haptoglobin* OR Helper Cell* OR Hematocrit* OR Hemoglobin* OR Hemopexin* OR Hpx OR hsCRP OR HVEML OR HVEM-L OR Hx OR Hydroxydeoxyguanosine* OR IFN OR IgE OR Il OR Il6 OR Inducer Cell* OR Inflammat* OR innate lymphoid cell* OR Intercrine* OR Interdigitating Cell* OR Interferon* OR Interleukin* OR Intraepithelial Lymphocyte* OR Isoferritin* OR Isotransferrin* OR Langerhans Cell* OR LE Cell* OR Leif OR Leucocyte* OR Leukocyte* OR Lymphocyte* OR Lymphotoxin* OR Macrophage* OR MAIT Cell* OR Marker* OR Mast Cell* OR Mastocyte* OR Monocyte* OR Natural Killer Cell* OR neutrophil* OR NK Cell* OR OPGL OR Orosomucoid* OR Osteoprotegerin Ligand OR OX40L OR Oxodeoxyguanosine* OR Oxoguanosine OR Pct OR Plasma cell* OR Plasmacyte* OR Platelet* OR procalcitonin* OR Prolastin OR RANK Ligand OR RANKL OR RbC OR RbCC OR Reticulocyte* OR Seromucoid* OR Serotransferrin* OR Serpin A1 OR Siderophilin* OR Soluble urokinase Plasminogen Activator Receptor* OR SPINK1 OR SuPAR OR THANK Protein OR Thymocyte* OR Thyrocalcitonin* OR tissue necrosis factor* OR TNF OR TNFa* OR Transferrin* OR Treg Cell* OR Tumor Necrosis Factor* OR Tumour Necrosis Factor* OR Veiled Cell* OR WbC OR WbCC OR Zemaira) |
| S4 | Animaux | MH(Animals+ NOT human) OR TI(rat OR rats OR mouse OR mice OR murine OR Guinea Pig*) OR AB(rat OR rats OR mouse OR mice OR murine OR Guinea Pig*) |
| S5 | Combinaison | (S1 AND S2 AND S3 AND LA(english OR French)) NOT S4 |

## Clarivate Web of Science

| #1 | Vitamine D | TS=(25OHD* OR 25OH-D* OR 25-OHD* OR 25-OH-D* OR dht intensol OR Dihydroxyvitamin-D* OR Dihydroxyvitamins-D* OR dnt intensol OR Hydroxyvitamin-D* OR Hydroxyvitamins-D* OR MC 1288 OR MC1288 OR vitamin-D* OR vitamins-D* OR antitanil OR antitetanin* OR atecen OR bocatriol OR calcamin* OR Calcidiol* OR Calcifediol* OR Calciferol* OR calcijex OR calcinosefaktor OR Calciol* OR calcitriol OR Calderol* OR Cholercalciferol* OR decostriol OR Dedrogyl* OR dichistrolum OR dichysterol OR dichystrol OR dihydral OR dihydrotachysterin* OR Dihydrotachysterol* OR dihydroxycholecalciferol* OR DihydroxyvitaminD* OR DihydroxyvitaminsD* OR dikystrol OR dygratyl OR Ercalcidiol* OR Ergocalciferol* OR Hidroferol* OR Hydroxycalciferol* OR Hydroxycholecalciferol* OR Hydroxyergocalciferol* OR HydroxyvitaminD* OR HydroxyvitaminsD* OR hytakerol OR manipal OR osteotriol OR parterol OR renatriol OR silkis OR sitriol OR Soltriol OR tachidon OR tachystin* OR tachystol OR tetilan OR tirocal OR vitaminD* OR vitaminsD*) |
| --- | --- | --- |
| #2 | Asthme | TS=Asthma* |
| #3 | Biomarqueurs d'inflammation | TS=(27E10 Antigen OR 4-1BB Ligand OR 4-1BBL OR 45S RNP OR A Proliferation Inducing Ligand Protein* OR Acid alpha 1-Glycoprotein* OR Acidic Pancreatic Trypsin Inhibitor* OR alpha 1 Antiprotease OR alpha 1 Antitrypsin OR alpha 1-Acid Glycoprotein* OR alpha 1-Antichymotrypsin* OR alpha 1-Antiproteinase OR alpha 1-Glycoprotein Acid* OR alpha 1-Macroglobulin* OR alpha 1-Protease Inhibitor OR alpha 1-Proteinase Inhibitor OR alpha 2-Globulin* OR alpha 2-Macroglobulin* OR alpha Macroglobulin* OR alpha-Macrofetoprotein* OR Amyloid A Precursor OR Amyloid A Protein OR Amyloid A OR Amyloid Fibril Protein AA OR Amyloid P Component* OR Amyloid Protein AA OR Amyloid Protein AA OR Amyloid Protein SAA OR Amyloid Serum Protein SAA OR Antigen L1 OR Antigen-Presenting Cell* OR Apo 2 Ligand OR Apo 3 Ligand OR Apo2 Ligand OR Apo3 Ligand OR APRIL Protein* OR B cell* OR B Lymphocyte Stimulator* OR B1 Cell* OR B-1 Cell* OR B1 Lymphocyte* OR B-1 Lymphocyte* OR B2 Cell* OR B-2 Cell* OR B2 Lymphocyte* OR B-2 Lymphocyte* OR B-Cell Activating Factor* OR Be1 Cell* OR Be2 Cell* OR beta-1 Metal-Binding Globulin OR beta-1B-glycoprotein OR B-Lymphocyte Activating Factor* OR B-Lymphocyte* OR B-Lymphoid* OR C3 Complement* OR C3 Precursor* OR Caeruloplasmin* OR CD 4 OR CD134 Ligand OR CD137 Ligand OR CD153 Antigen* OR CD154 Antigen* OR CD178 Antigen* OR CD253 Antigen* OR CD254 Antigen* OR CD257 Antigen* OR CD30 Ligand OR CD40 Ligand OR CD4-Positive Lymphocyte* OR CD54 Antigen* OR CD70 Antigen* OR CD8 Positive Lymphocyte* OR CD95 Ligand OR Complement 3 OR Complement C3 OR Complement Component 3 OR COX 2 OR D dimer OR D1 dimer OR E Selectin* OR ELAM* OR Endothelial Leukocyte Adhesion Molecule-1 OR Fas Ligand OR fibrin fragment D OR Herpesvirus Entry Mediator-Ligand Protein* OR ICAM* OR Immunoglobulin E OR Immunologic Accessory Cell* OR Intercellular Adhesion Molecule* OR Kazal Pancreatic Trypsin Inhibitor* OR L1 Antigen OR LAK Cell* OR LECAM-2 OR LIGHT Protein* OR Lipocalin-2 OR Lymphokine Activated Killer Cell* OR Neutrophil Gelatinase-Associated Lipocalin OR NGAL Protein* OR Oncogene 24p3 Protein* OR Osteoclast Differentiation Factor* OR OX40 Ligand OR Pancreatic Secretory Trypsin Inhibitor* OR Pro-C3 OR Receptor Activator of Nuclear Factor-kappa* OR Serpin A3 OR Serum A Related Protein OR Serum Amyloid A OR Serum Amyloid P OR Serum Amyloid Protein A OR Serum Sialomucin* OR Siderocalin Protein* OR T cell* OR T Helper OR T Lymphocyte* OR T4 cell* OR T4 Lymphocyte* OR T8 Cell* OR T8 Lymphocyte* OR TALL-1 Protein* OR TALL2 Protein* OR TALL-2 Protein* OR TC1 Cell* OR TC2 Cell* OR Tfh Cell* OR Th1 Cell* OR Th17 Cell* OR Th2 Cell* OR Th3 Cell* OR TL1 Ligand OR TL1A Ligand OR T-Lymphoid* OR TNFS* OR Tr1 Cell* OR TRAIL Protein* OR TRANCE Protein* OR Trypsin Inhibitor Kazal Pancreatic OR Tumor Associated Trypsin Inhibitor* OR tumor necrosis serum* OR tumour necrosis serum* OR Vascular Cell Adhesion Molecule* OR VCAM* OR 8OHdG OR 8-OH-dG OR 8-oxodG OR 8-oxo-dG OR 8-oxodGuo OR 8-oxo-dGuo OR A1PI OR acute phase globulin* OR Acute Phase Glycoprotein* OR acute phase plasma protein* OR acute phase protein* OR Acute Phase Reactant* OR acute phase response protein* OR Amyloid Protein* OR Apo2L OR Apo2-L OR Apo-2-L OR Apo3L OR Apo3-L OR Apo-3-L OR Apoferritin* OR BAFF Ligand OR Basophil* OR Biomarker* OR Blood cell* OR Blood Count* OR Blood Number* OR Bloodcell* OR BLyS Protein OR Breg Cell* OR C Reactive Protein OR Cachectin OR Cachetin OR Calcitonin* OR Calcitrin* OR Calgranulin* OR Calprotectin* OR CD134L OR CD27 Ligand OR CD27L OR CD30L OR CD4 OR CD40L OR CD62E OR CD95L OR Cell Count* OR Cell Number* OR Ceruloplasmin* OR Chemokine* OR Ciba 47175* OR Ciba47175* OR COX2 OR CRP OR CycloOxygenase* OR Cyclo-Oxygenase* OR Cytokine* OR Dendritic Cell* OR Deoxyguanosine* OR Dihydrodeoxyguanosine* OR Dihydroguanosine* OR Ectodysplasin* OR eNO OR Eosinophil* OR Eosinophil* OR Erythrocyte* OR ESR OR Exhaled Nitric Oxide* OR exhaled NO OR Feno OR Ferritin* OR Ferroxidase* OR Fibrinogen* OR Granulocyte* OR Haemopexin* OR Haptoglobin* OR Helper Cell* OR Hematocrit* OR Hemoglobin* OR Hemopexin* OR Hpx OR hsCRP OR HVEML OR HVEM-L OR Hx OR Hydroxydeoxyguanosine* OR IFN OR IgE OR Il OR Il6 OR Inducer Cell* OR Inflammat* OR innate lymphoid cell* OR Intercrine* OR Interdigitating Cell* OR Interferon* OR Interleukin* OR Intraepithelial Lymphocyte* OR Isoferritin* OR Isotransferrin* OR Langerhans Cell* OR LE Cell* OR Leif OR Leucocyte* OR Leukocyte* OR Lymphocyte* OR Lymphotoxin* OR Macrophage* OR MAIT Cell* OR Marker* OR Mast Cell* OR Mastocyte* OR Monocyte* OR Natural Killer Cell* OR neutrophil* OR NK Cell* OR OPGL OR Orosomucoid* OR Osteoprotegerin Ligand OR OX40L OR Oxodeoxyguanosine* OR Oxoguanosine OR Pct OR Plasma cell* OR Plasmacyte* OR Platelet* OR procalcitonin* OR Prolastin OR RANK Ligand OR RANKL OR RbC OR RbCC OR Reticulocyte* OR Seromucoid* OR Serotransferrin* OR Serpin A1 OR Siderophilin* OR Soluble urokinase Plasminogen Activator Receptor* OR SPINK1 OR SuPAR OR THANK Protein OR Thymocyte* OR Thyrocalcitonin* OR tissue necrosis factor* OR TNF OR TNFa* OR Transferrin* OR Treg Cell* OR Tumor Necrosis Factor* OR Tumour Necrosis Factor* OR Veiled Cell* OR WbC OR WbCC OR Zemaira) |
| #4 | Animaux | TS=(rat OR rats OR mouse OR mice OR murine OR Guinea Pig*) |
| #5 | Combinaison | (#1 AND #2 AND #3) NOT #4  Refined By:Languages: French or English |

# Supplementary Figures


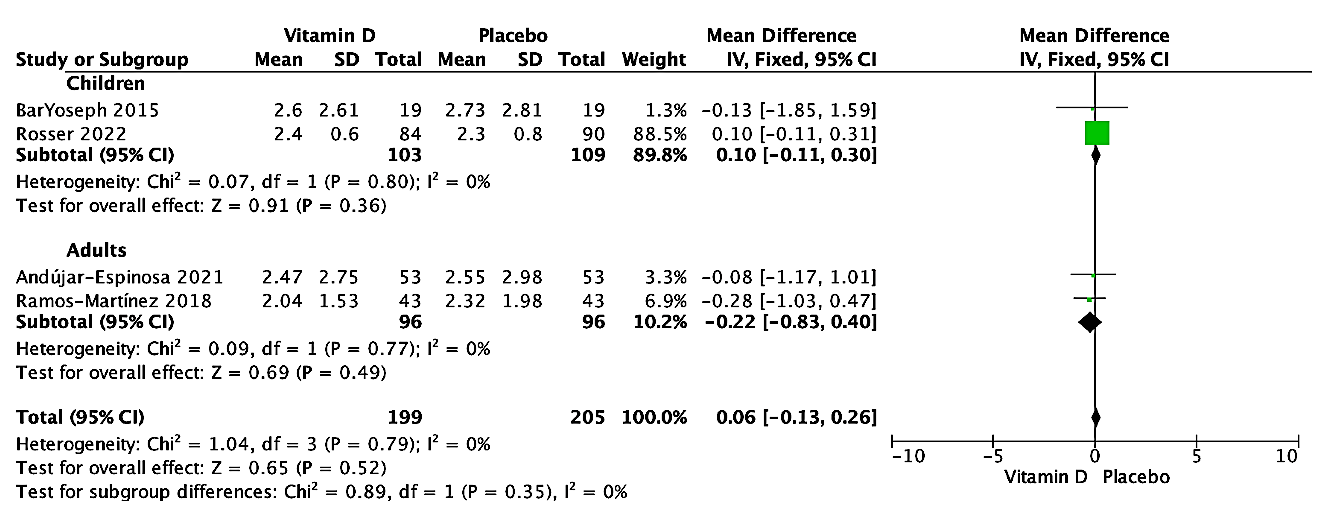


**Figure S1.** Forest plot of randomized controlled trials investigating the effects of vitamin D supplementation on serum total IgE (IU/mL) by age group. Mean group differences in a log scale are presented with 95% CIs and calculated with the fixed-effects model. Heterogeneity was quantified by I^2^ at a significance of P < 0.10.


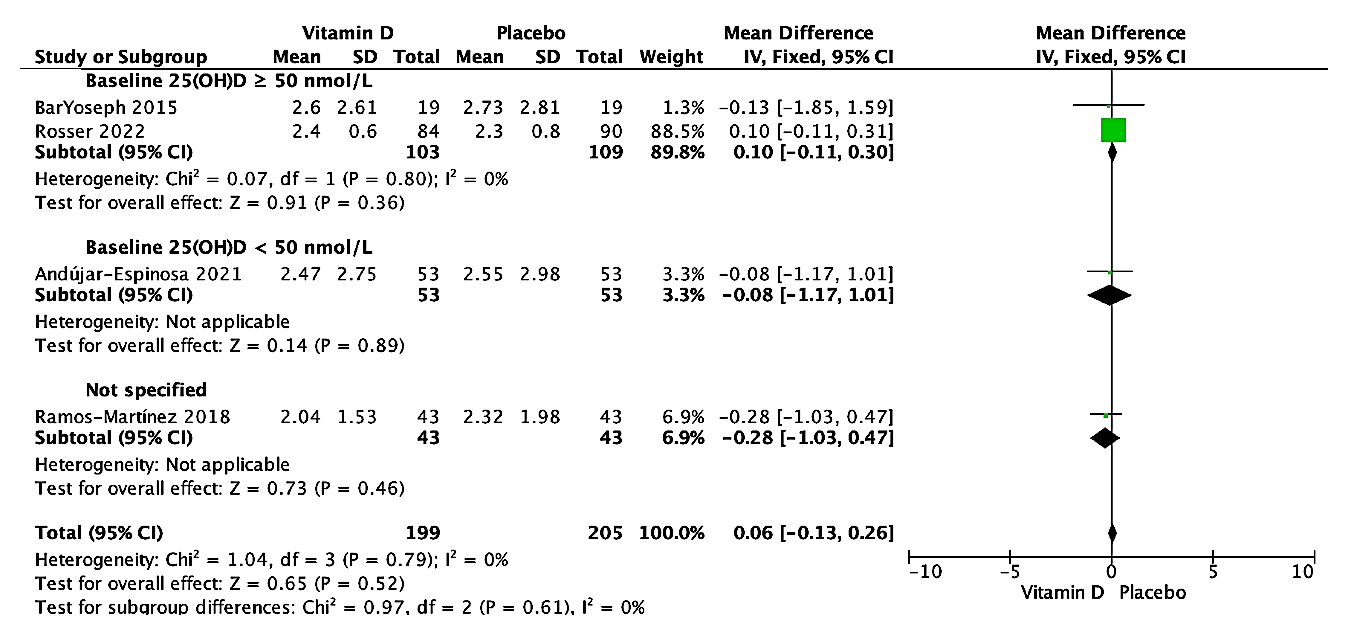


**Figure S2**. Forest plot of randomized controlled trials investigating the effects of vitamin D supplementation on serum total IgE (IU/mL) by vitamin D status group. Mean group differences in a log scale are presented with 95% CIs and calculated with the fixed-effects model. Heterogeneity was quantified by I^2^ at a significance of P < 0.10.


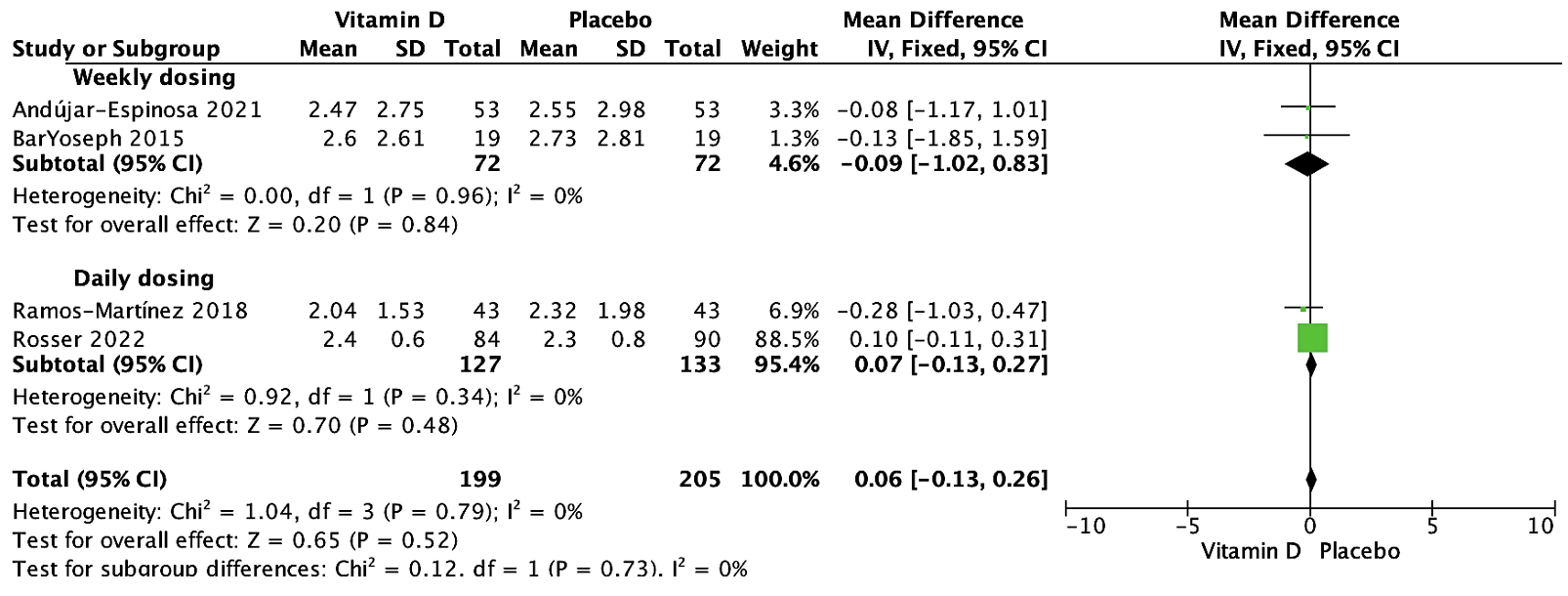


**Figure S3**. Forest plot of randomized controlled trials investigating the effects of vitamin D supplementation on serum total IgE (IU/mL) by dosing regimen. Mean group differences in a log scale are presented with 95% CIs and calculated with the fixed-effects model. Heterogeneity was quantified by I^2^ at a significance of P < 0.10.


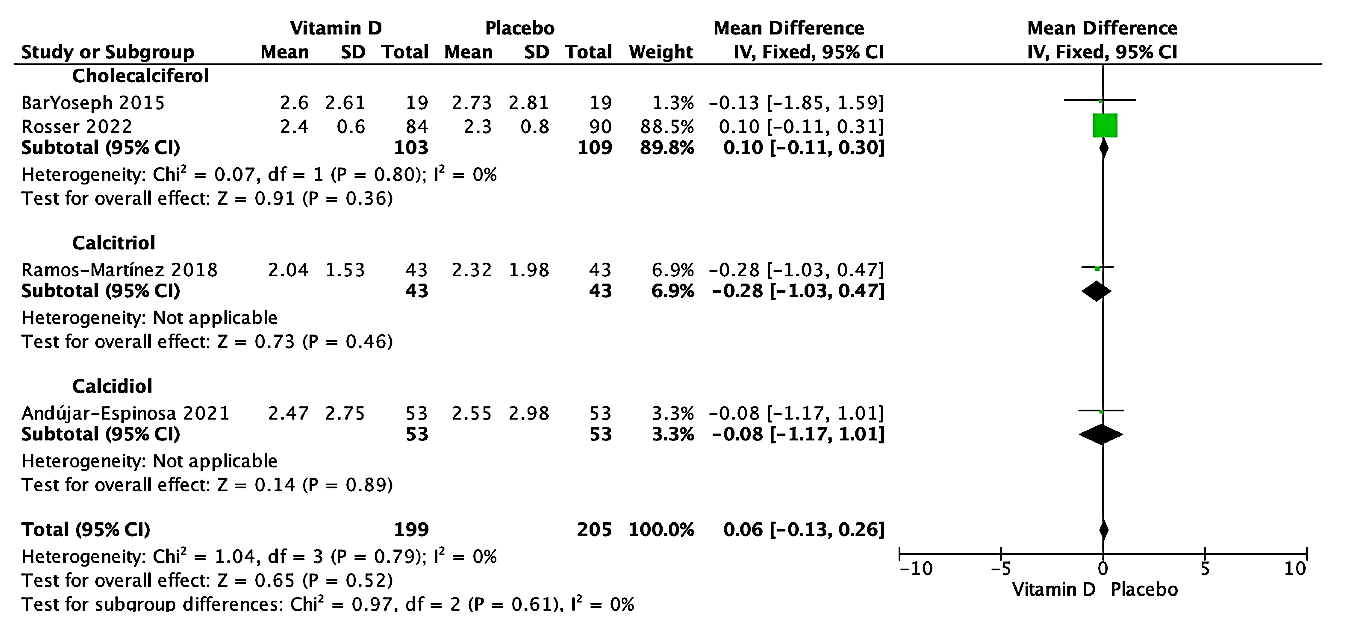


**Figure S4**. Forest plot of randomized controlled trials investigating the effects of vitamin D supplementation on serum total IgE (IU/mL) by vitamin D form. Mean group differences in a log scale are presented with 95% CIs and calculated with the fixed-effects model. Heterogeneity was quantified by I^2^ at a significance of P < 0.10.

**
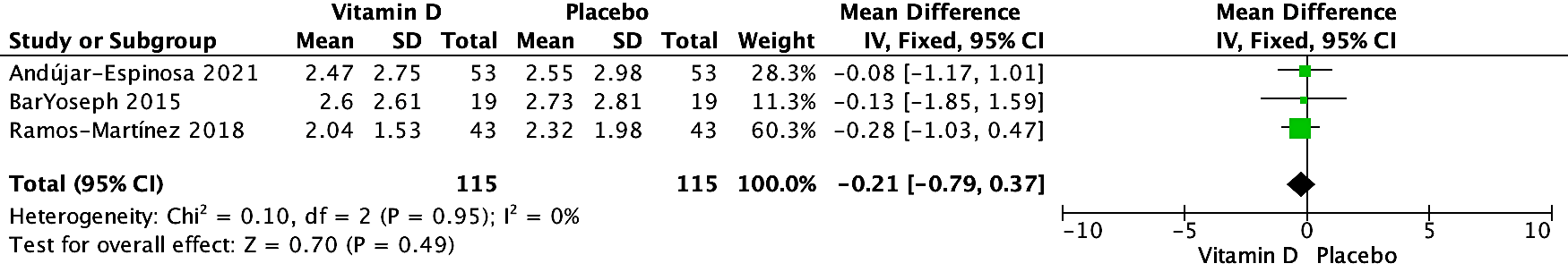
**

**Figure S5**. Forest plot of randomized controlled trials investigating the effects of vitamin D supplementation on serum total IgE (IU/mL) after excluding studies with unclear risk of bias. Only studies with a low risk of bias were included. Mean group differences in a log scale are presented with 95% CIs and calculated with the fixed-effects model. Heterogeneity was quantified by I^2^ at a significance of P < 0.10.


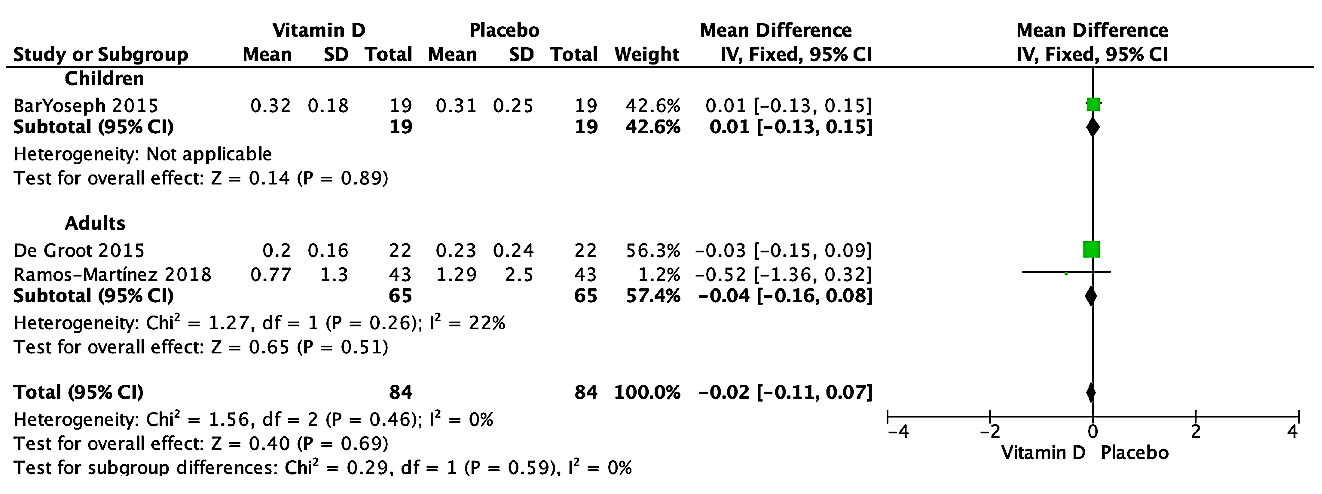


**Figure S6**. Forest plot of randomized controlled trials investigating the effects of vitamin D supplementation on blood eosinophils (10^3^/μL) by age group. Values are mean differences with 95% CIs determined with the use of fixed-effects model. Heterogeneity was quantified by I^2^ at a significance of P < 0.10.


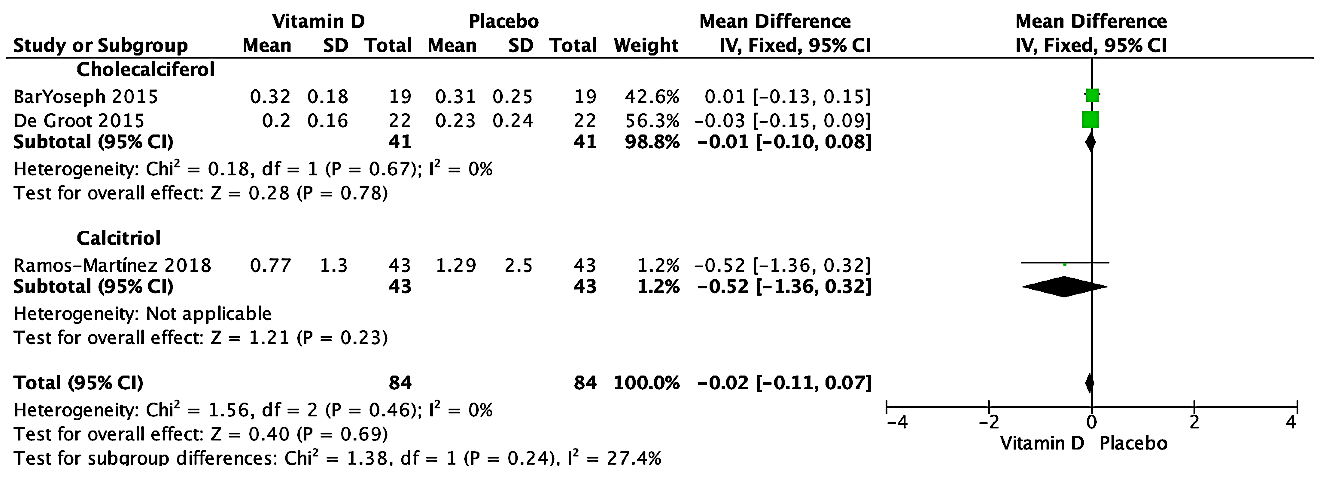


**Figure S7**. Forest plot of randomized controlled trials investigating the effects of vitamin D supplementation on blood eosinophils (10^3^/μL) by vitamin D form. Values are mean differences with 95% CIs determined with the use of fixed-effects model. Heterogeneity was quantified by I^2^ at a significance of P < 0.10.


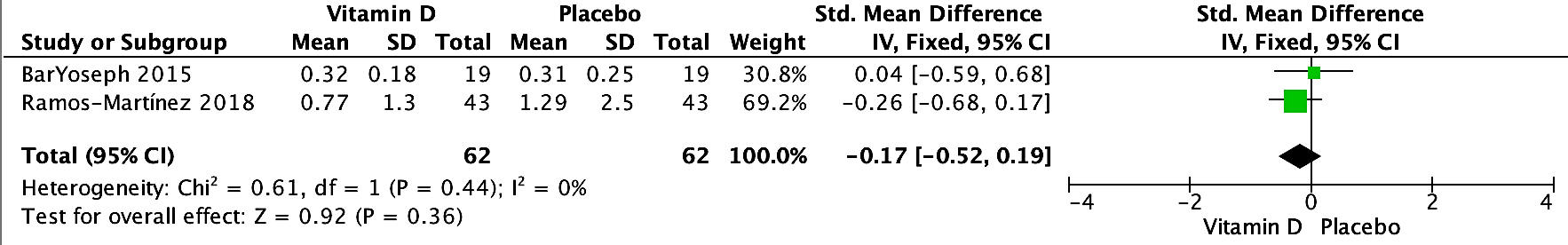


**Figure S8**. Forest plot of randomized controlled trials investigating the effects of vitamin D supplementation on blood eosinophils (10^3^/μL) after excluding studies with initial results reported as median (IQR). Values are mean differences with 95% CIs determined with the use of fixed-effects model. Heterogeneity was quantified by I^2^ at a significance of P < 0.10. IQR, interquartile range.


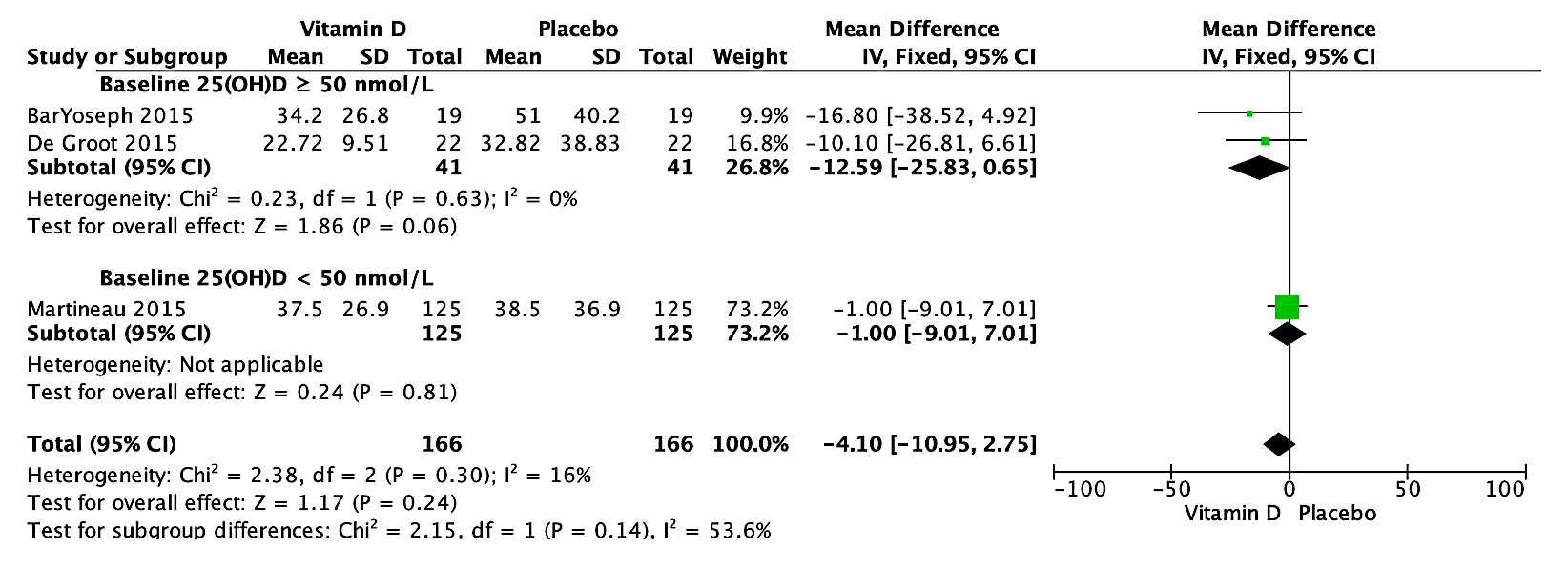


**Figure S9**. Forest plot of randomized controlled trials investigating the effects of vitamin D supplementation on FeNO (ppb) by vitamin D status group. Values are mean differences with 95% CIs determined with the use of fixed-effects model. Heterogeneity was quantified by I^2^ at a significance of P < 0.10.


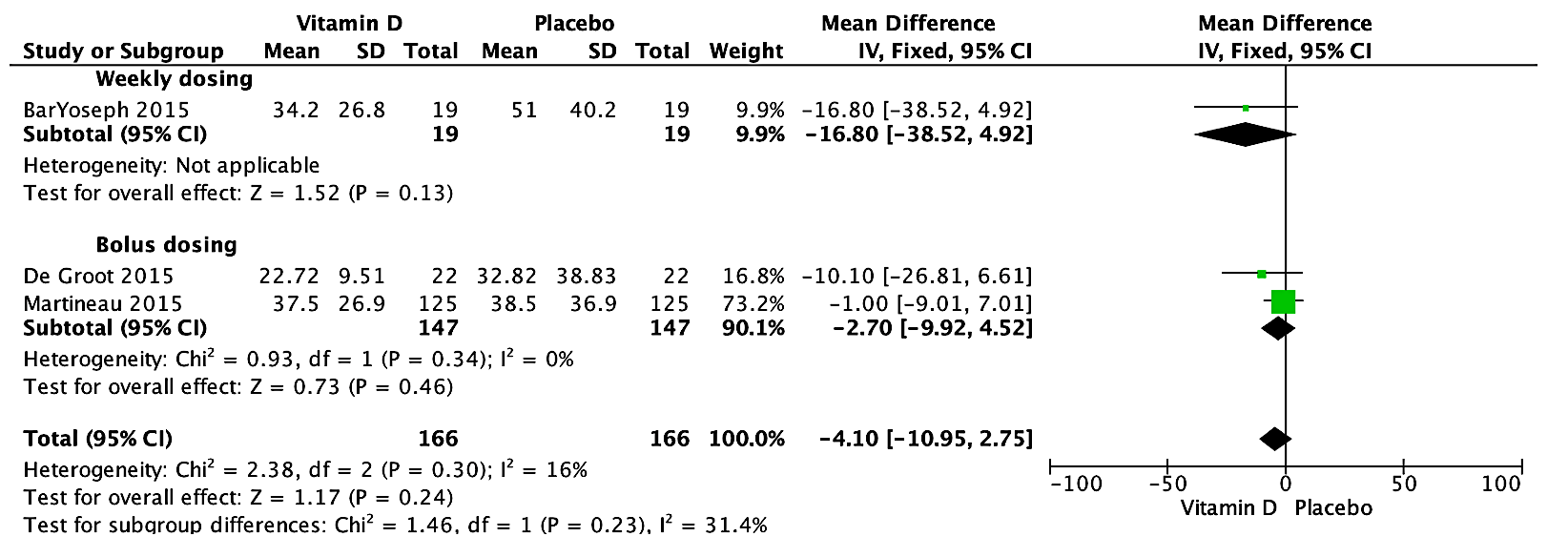


**Figure S10.** Forest plot of randomized controlled trials investigating the effects of vitamin D supplementation on FeNO (ppb) by dosing regimen. Values are mean differences with 95% CIs determined with the use of fixed-effects model. Heterogeneity was quantified by I^2^ at a significance of P < 0.10.


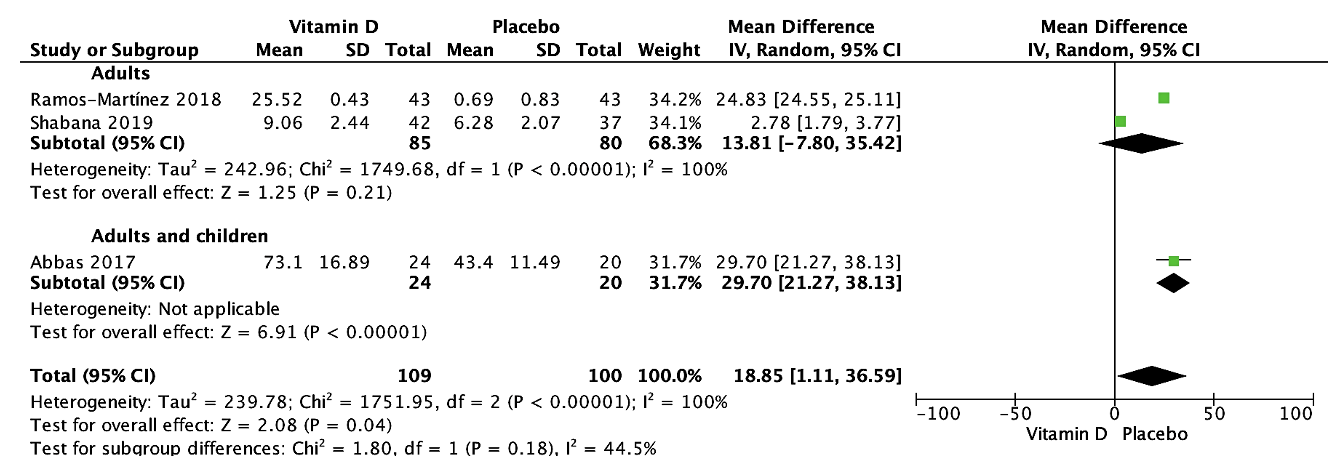


**Figure S11**. Forest plot of randomized controlled trials investigating the effects of vitamin D supplementation on serum IL-10 (pg/mL) by age group. Values are mean differences with 95% CIs determined with the use of random-effects model. Heterogeneity was quantified by I^2^ at a significance of P < 0.10.


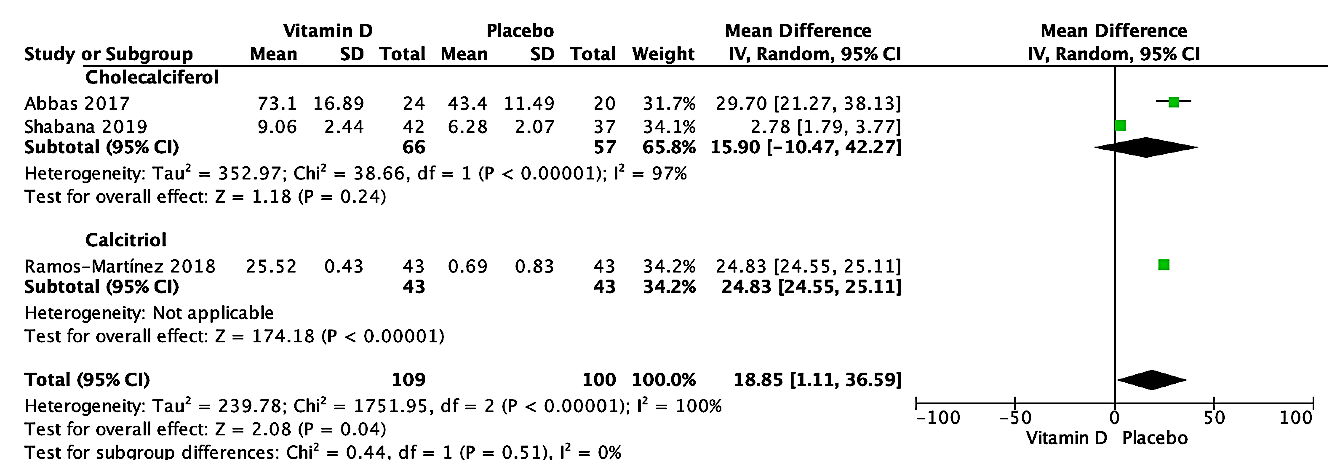


**Figure S12**. Forest plot of randomized controlled trials investigating the effects of vitamin D supplementation on serum IL-10 (pg/mL) by vitamin D form. Values are mean differences with 95% CIs determined with the use of random-effects model. Heterogeneity was quantified by I^2^ at a significance of P < 0.10.
